# Development of New Materials with Different Types of By-Products to Manufacture Construction Elements for Thermal Insulation

**DOI:** 10.3390/ma19204409

**Published:** 2026-10-20

**Authors:** Ali Musa, Leena Ahmed, Manuel Rosales, Francisco Agrela, Julia Rosales

**Affiliations:** 1 Construction Engineering Area, University of Córdoba, 14071 Córdoba, Spain; z42mumua@uco.es (A.M.); z32ahahl@uco.es (L.A.); mrosales@uco.es (M.R.); fagrela@uco.es (F.A.); 2 Department of Civil Engineering, Tobruk University, Tobruk, Libya; 3 Area of Mechanics of Continuous Media and Theory of Structures, University of Cordoba, 14071 Córdoba, Spain

**Keywords:** sustainable mortars, biomass bottom ash, mixed recycled aggregates, chamotte, wood waste, thermal conductivity, mechanical behavior

## Abstract

**Highlights:**

• By-products were assessed as partial cement and sand substitutes in sustainable thermal-insulating mortars.
• Chamotte as a cement replacement improved strength, reaching 41% higher compressive strength than the reference at 90 days.
• MRA as a cement replacement and expanded clay provided a balanced reduction in density, thermal conductivity, and mechanical losses.
• The optimized mortar reached ~39 MPa and reduced thermal conductivity by nearly 22% versus the reference.

**Abstract:**

This study evaluates mortars that incorporate mixed recycled aggregates, biomass bottom ash, chamotte, wood waste, and expanded clay as partial substitutes for cement and natural aggregates. Three series of mortars were investigated: with 25% cement replacement, partial sand replacement, and simultaneous replacement of cement and sand. Mechanical, physical, durability, and thermal properties were evaluated, along with their relationships through a correlation analysis. Cement substitution did not result in a drastic reduction in compressive strength and produced limited thermal improvements, whereas sand substitution led to substantially greater reductions in thermal conductivity, ranging from approximately 10.3% for MRA to 59.1% for wood waste compared to the control. Expanded clay reduced thermal conductivity by 24.7%, while BBA-L showed greater insulating potential than BBA-PG but resulted in a reduction in compressive strength. The combination of chamotte, limestone filler as a cement substitute, MRA, and expanded clay as an aggregate substitute provided the best balance between thermal and mechanical performance, achieving a compressive strength of approximately 39 MPa at 90 days and reducing thermal conductivity by nearly 22% compared to the reference mortar. The results demonstrate that thermal performance depends largely on density and porosity, although the optimal selection of materials requires simultaneously considering both mechanical and physical properties.

## 1. Introduction

The increase in the consumption of raw materials worldwide raises concerns about the environmental impact of CO_2_ emissions from producing and preparing these materials. The construction sector accounts for approximately 6 billion tons of CO_2_ emissions [1,2]. In Spain in 2010, CO_2_ per capita production was 5.8 metric tons, and in Europe, it was 7.4 metric tons.

The increasing demand for raw materials, such as aggregates, represents 700 million tons annually in Brazil, 900 million tons annually in the USA, and 2.7 billion tons annually in the European region [1]. In 2018, sand and gravel consumption in the European Union (EU) approached 2.25 billion tons. Since 2015, annual consumption has consistently exceeded 2 billion tons and continues to rise [3]. An estimated 453 million tons of aggregate were consumed in France alone in 2019 [4]. In 2021, around 4.3 billion tons of cement were produced in China, accounting for 55% of global consumption, while India consumed about 8% [5]. The increase in the consumption of raw materials can lead to a reduction in their restoration and an increase in CO_2_ emissions.

The construction sector is one of the biggest consumers of natural resources. It significantly contributes to environmental impacts, especially through cement production and natural aggregate extraction. In recent years, more focus has been placed on creating environmentally friendly building materials, especially mortars, which can be achieved by partially or totally replacing traditional raw materials with construction and demolition waste (CDW) and industrial byproducts. The amount of CDW generated yearly reaches 10,000 million tons, which corresponds to about 30–40% of all solid waste in the world [6]. The annual production of CDW in China is approximately 1.5 billion tons, while in Europe, it is around 850 million tons yearly. In Brazil, in 2016, approximately 45 million tons were produced [1]. This huge amount of CDW raises significant concerns about how to dispose of this waste. The quantity of waste materials suggests that they could be used in construction work. In general, some waste materials can offer significant advantages as replacements for cement or aggregate in producing different types of concrete, such as their light weight, thermal insulation, low cement content, etc. [5,7,8,9,10,11]. The primary waste materials that can provide such properties are wood, glass, and crushed bricks [7].

Another sector that generates large amounts of waste is the industrial sector, including energy production through biomass burning. This produces a waste product. Ash is a solid waste product produced when forest biomass is burned. Nowadays, the majority of the biomass ash generated by thermal power plants is either recycled on agricultural or forest lands or dumped in landfills, and both practices typically go unchecked [12]. In 2017, the estimated annual biomass bottom ash (BBA) product amounts in France and worldwide were about 200,000 tons and 170 million tons, respectively [4]. In addition, the waste generated by power generation products, approximately 120,000 tons of biomass in Spain, raises concerns about how to dispose of it and whether it can be utilized in construction materials [13]. According to a study, it is estimated that in 2100, biomass will be utilized to produce 25% to 46% of energy, which can be considered clean energy [14].

Currently, biomass accounts for about 15% of electricity production [15].

Biomass currently accounts for more than 10% of electricity supply and is regarded as the most significant renewable energy source. Despite its advantages, concerns remain regarding the disposal of residual byproducts [14,16,17,18].

The most popular strategy to reduce the environmental effect of cement production is to adopt alternative supplementary cementitious materials (SCM). Modest clinker cements are rarely used and are only utilized for applications with modest mechanical demands, whereas normal cements can have clinker contents ranging from 5% to 100% [19]. The utilization of fly ash and bottom ash from burning biomass as cement substitutes in mortar formulations was assessed and could be used as a cement replacement [20,21]. In addition, there is a study that demonstrated that the flexural strength was more influenced by both fine and coarse bottom ash particles than the compressive strength [22]. The use of biomass bottom ash in the production of mortars as a partial substitute for aggregates also leads to modifications in the mortar, such as a reduction in density or improved acoustic insulation properties.

Another type of industry that generates large amounts of waste is wood production. The wood industry produces a lot of waste materials globally. When a piece of wood is chopped from logs or by drilling, milling, and cutting processes, sawdust is produced, or while final items are being prepared. Wood dust, which is made up of extremely tiny particles created during sanding or other machining operations, is distinct from wood ash, which is the residue left behind after burning wood and its byproducts. It is frequently gathered in dust collectors or filter bags [7]. Looking at the overall picture of industrial waste production and the need to cut down on natural resources in making building materials.

The main objective of this research is to classify mortars manufactured with a high percentage of mixed recycled aggregates (MRA) from construction and demolition waste, forest biomass and olive tree waste, chamotte and wood as a substitute for cement and natural aggregates based on their physical properties and technologies, as well as to evaluate their thermal behavior for future application in building elements. The study aims to identify the optimal mixtures suitable for sustainable building element applications. Exhaustive studies have been carried out on MRA, BBA and chamotte separately in cement-based systems. However, their combined application, especially in mortar formulations, remains largely unexplored. Currently, there is no precise classification system to evaluate and standardize the technical performance of mortars containing these by-products for use in building. Misunderstandings among stakeholders, caused by differences in performance and the lack of legal requirements, prevent the widespread use of eco-friendly mortars.

The aim of this study is to evaluate the properties of mortars, including those made from a combination of these by-products, and to compare them with a reference mortar. A study of their short- and medium-term technical properties will be carried out, along with an assessment of the influence on the thermal properties of the mortars. This study will form the basis for demonstrating the feasibility of using these by-products in various building components.

## 2. Materials

In this study, various materials were used to manufacture mortars. First, the conventional materials used were analyzed, which were Portland cement type Cem I, limestone filler, sand and expanded clay.

As for the by-products used as alternative materials, olive biomass bottom ash (BBA-PG) and forest pruning residues (BBA-L), mixed recycled aggregates (MRA), wood waste (W) and residual chamotte (CH) were studied.

The by-products were subjected to two processing systems.

- Processing of material for use as a partial substitute for aggregate. First, they underwent crushing and screening processes to obtain a particle size similar to that of natural sand (0–2 mm) and were used as a partial aggregate substitute. This treatment was applied to the bottom ash from biomass from Puente Genil, Córdoba, Spain (BBA-PG), bottom ash from biomass from Linares (BBA-L), and mixed recycled aggregate (MRA). The wood waste (W) was shredded and screened until a particle size of 0–4 mm was achieved.
- Processing of material for use as a partial substitute for cement. Second, pulverization treatments were applied to use the materials as a partial cement substitute (SCM). The by-products that were pulverized and sieved through a screen with a mesh size of less than 0.125 mm for use as a partial cement substitute were Linares Biomass Bottom Ash from Linares, Córdoba, Spain, Mixed Recycled Aggregate and Chamotte, designated pBBA-L, pMRA and pCH, respectively.

**Figure 1:**
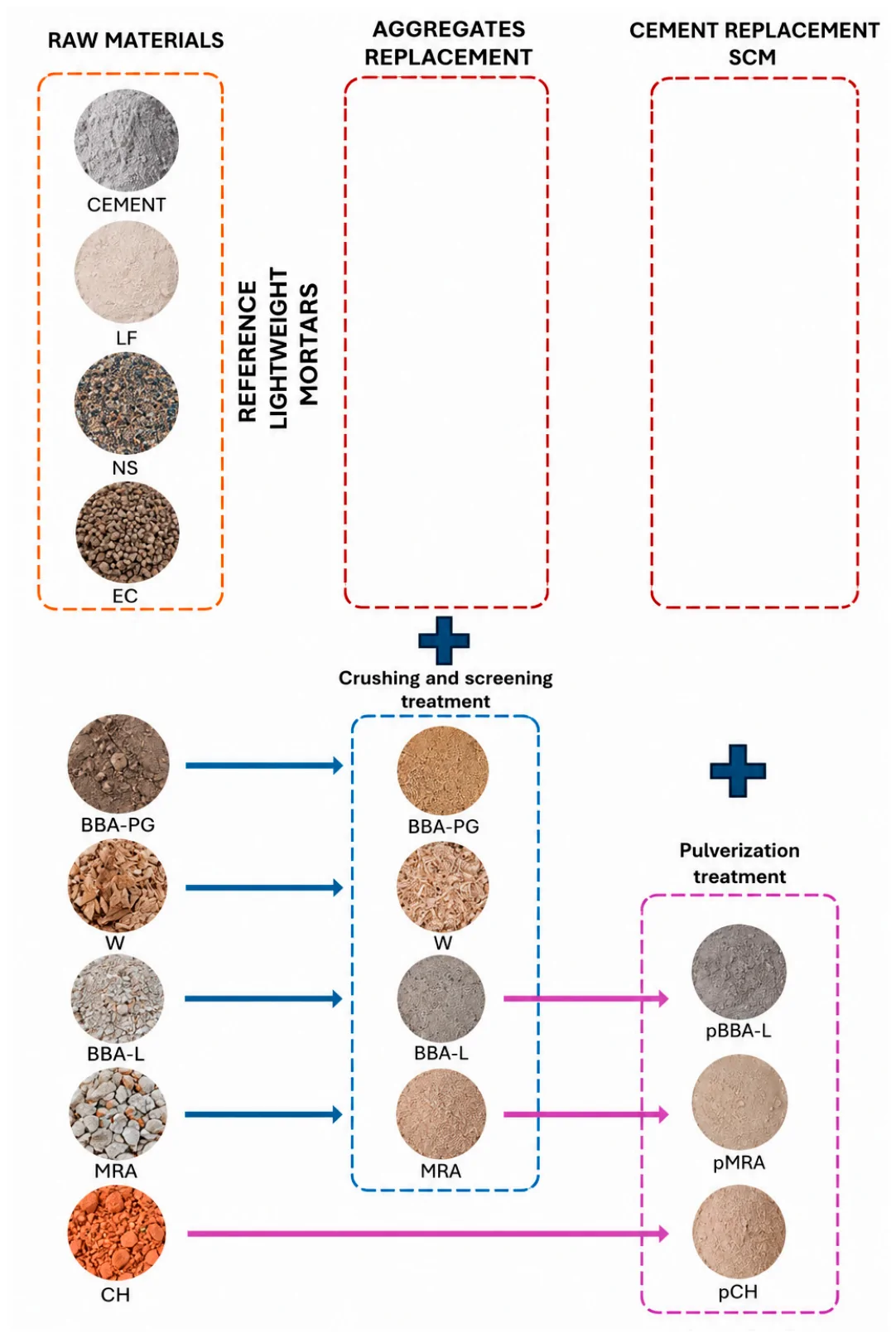
Materials used and processed.

Figure 1 shows the materials used as partial substitutes for aggregate or cement and the appearance of each of them after undergoing the different processing methods.

Below is a detailed description of each of the materials and their physical and chemical properties, together with a leaching study to assess the environmental impact that their application may cause.

### 2.1. Conventional Raw Materials

#### 2.1.1. Cement

Ordinary Portland cement (CEM-I 52.5 N, from Votorantim Cimentos, Niebla, Huelva, Spain) was utilized in this investigation. This high-resistance cement contains almost 95% clinker. It achieves a 28-day compressive strength of 52.5 MPa. The chemical composition of OPC, summarized in Table 1, was measured by wavelength-dispersive X-ray fluorescence (XRF) spectrometry, following UNE EN 196-2 [23].

#### 2.1.2. Limestone Filler (LF)

In this work, Omya Clariana S.L.U. (Darro, Spain) provided the calcium carbonate. High-purity ground calcium carbonate (CaCO_3_) accounted for the majority of the material. It has a fine particle size distribution, chemical stability, and whiteness from natural limestone deposits. These features allow its use as mineral fillers or additives in cementitious and polymer-based composites. Table 2 shows the material properties.

#### 2.1.3. Natural Sand (NS)

**Figure 2:**
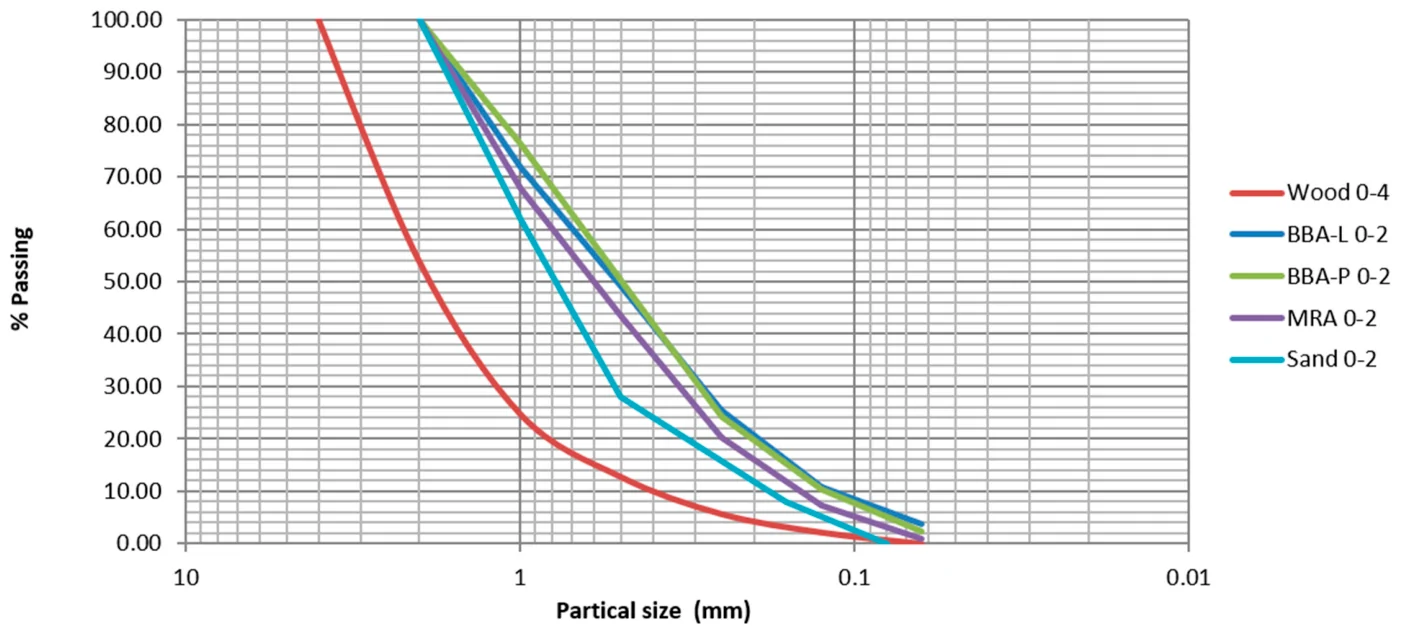
Particle size distribution of materials used as a partial aggregate substitute.

The distribution of particle sizes in the normalized natural sand is specified in DIN EN 196-1 [29], as shown in Figure 2. The sand used for this project is standard sand with a size range of 0.08 to 2 mm. The density and maximum moisture content were 2.57 g/cm^3^ and 0.18%, respectively (Table 2), similar to the results obtained in previous works [30].

#### 2.1.4. Expanded Clay (EC)

A lightweight aggregate made from expanded clay (EC) was used. EC has an apparent density of 350 kg/m^3^, water absorption of 34%, and a size of between 2 and 10 mm. The characteristics of EC are shown in Table 2.

### 2.2. Alternative By-Product Materials as Partial Substitutes for Aggregates

Biomass Bottom Ash (BBA), Mixed Recycled Aggregate (MRA), and Wood Chip Waste (W) were evaluated as partial substitutes for aggregates in the manufacture of mortars. The properties of each of these materials are described below.

#### 2.2.1. Biomass Bottom Ash (BBA)

Two types of biomass bottom ash were utilized in this study as replacements for cement and fine aggregates. In this section, we will discuss the properties of the materials used to replace aggregates. The two types of biomass originate from an electricity plant located in Linares (BBA-L) and Puente Genil (BBA-PG), both in Córdoba. The biomass mainly consists of 40% remaining 40% olive cake and 60% wood [31,32].

BBA-L and BBA-PG mainly consist of silica, aluminum oxide, and quicklime, which have a chemical composition as shown in Table 3, which is similar to Rosales et al., 2023 results [30]. According to other investigations, the primary oxides identified in the ash were CaO, SiO_2_, and Al_2_O_3_, which are also the main ingredients in Portland cement [15]. Except for sugarcane ash, which is rich in SiO_2_, the two most abundant elements in the majority of biomass ash types are CaO, SiO_2_, or K_2_O [15]. This implies that biomass ash may be more broadly classified according to the relative abundances of CaO, K_2_O, and SiO_2_ [15].

The biomass used as a replacement for sand was sieved to obtain a distribution of 0–2 mm, as shown in Figure 2. The particle size distribution is like that of natural sand. It contains a significant proportion of fine particles and has a relatively uniform distribution. With respect to density and water absorption, the biomass has a low density compared to natural sand for BBA-L and BBA-PG, which are 1.897 and 2.311 Kg/m^3^, respectively. The water absorption and density values are comparable to those in previous studies [14,33], which indicates a lower density than sand, at 2.55 Kg/m^3^.

**Figure 3:**
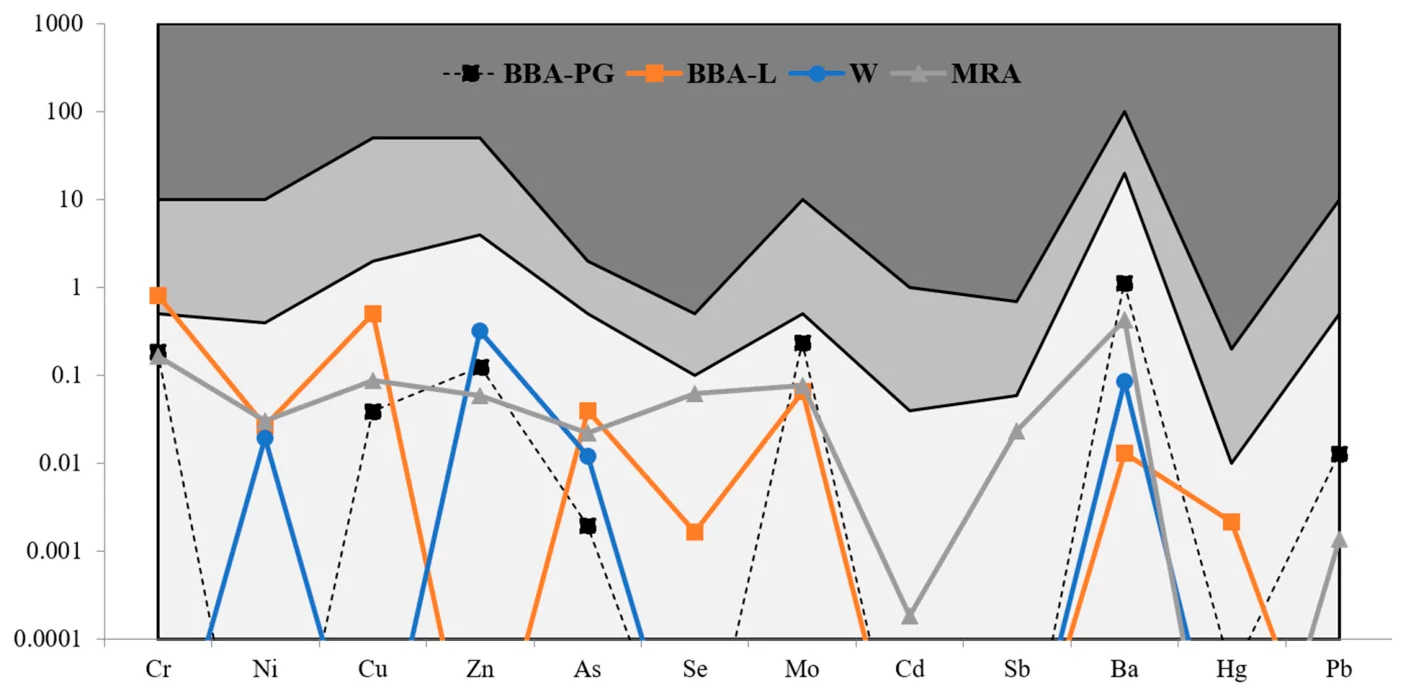
Analysis of heavy metal leaching from materials used as a partial substitute for aggregate. Classification limits in accordance with European Council Decision 2003/33/EC are shown in the graph. Light gray indicates inert material, medium gray indicates non-hazardous material, and dark gray indicates hazardous material.

In addition, a leaching study was carried out to assess the environmental hazard posed by aggregates. The BBA leachates were examined using the European standard UNE-EN 12457-4 [34]. To classify BBA as inert, non-hazardous, or hazardous waste, the leachate concentration of components was determined using an L/S ratio of 10 L/kg in compliance with European Council Decision 2003/33/EC [35]. The values obtained for the analyzed components are presented in Figure 3, where they are compared with the defined regulatory limits. Cr for BBA-L is the sole element that is above the inert waste limit, as can be observed. The value is right on the limit and classifies the material as non-hazardous; previous experience has shown that when it is incorporated as an aggregate into a cementitious matrix, it becomes encapsulated and the leachate is considered inert [36].

#### 2.2.2. Mixed Recycled Aggregates (MRA)

In this study, MRA, manufactured by Gecorsa company based in Córdoba, Spain was used as two different materials. It is used as a sand substitute after being sieved to a size of 0–2 mm, as illustrated in Figure 2. The density and absorption values are 2.38 Kg/m^3^ and 4.7%, respectively, which is consistent with most standards [37]. The main components of the aggregate are ceramic, concrete, and aggregate. Its physical properties are shown in Table 2. Furthermore, the chemical compositions, determined by X-ray fluorescence (XRF), are also presented in Table 4.

The chemical composition primarily depends on the origin of the samples [30]. The chemical composition of the MRA sample is dominated by oxides of calcium (CaO, 40.48%) and silicon (SiO_2_, 36.57%), which together account for around 77% of the total composition. This high proportion indicates that the material is predominantly calcosilicate in nature, a characteristic of cementitious materials, slags, or certain industrial wastes.

The third most abundant component is aluminum oxide (Al_2_O_3_, 9.04%), the presence of which may promote the formation of aluminosilicate phases and contribute to the material’s reactivity. Iron oxide (Fe_2_O_3_, 3.73%) and magnesium oxide (MgO, 2.89%) are present in moderate proportions and are commonly found in mineral and cementitious materials.

The presence of SO_3_ (3.33%) is relatively significant and may be associated with sulphate compounds, a factor that should be taken into account due to its influence on hydration processes and the durability of the material. The alkali oxides, K_2_O (1.90%) and Na_2_O (1.09%), are present in low concentrations, although sufficient to influence the alkalinity of the system and, in some cases, reactions such as alkali-silica.

In addition, to assess the potential use of the material as a partial substitute for aggregate, an environmental impact study was carried out by analyzing leachate (Figure 3). The material was classified as inert and would therefore not pose any environmental problems.

#### 2.2.3. Wood (W)

The Onesta company in Montilla, Cordoba, Spain supplied wood chips. The wood underwent several processes to achieve the desired distribution size, ranging from 0 to 4 mm. The wood was treated for two hours with a 2.5% NaOH solution (WT) at room temperature 20 ± 2 °C then washed several times with tap water until the pH measured 7. The density of the wood was measured before and after treatment, and was 0.96 and 1.11 g/cm^3^, respectively. The wood has irregular shapes and is used as a sand replacement to produce lightweight mortar. Like the other materials, its environmental properties were analyzed to determine whether the material presents any issues regarding its use as a partial substitute for aggregate (Figure 3), and it was found that the material is classified as inert.

### 2.3. Alternative By-Products Materials as Partial Substitutes for Cement

**Figure 4:**
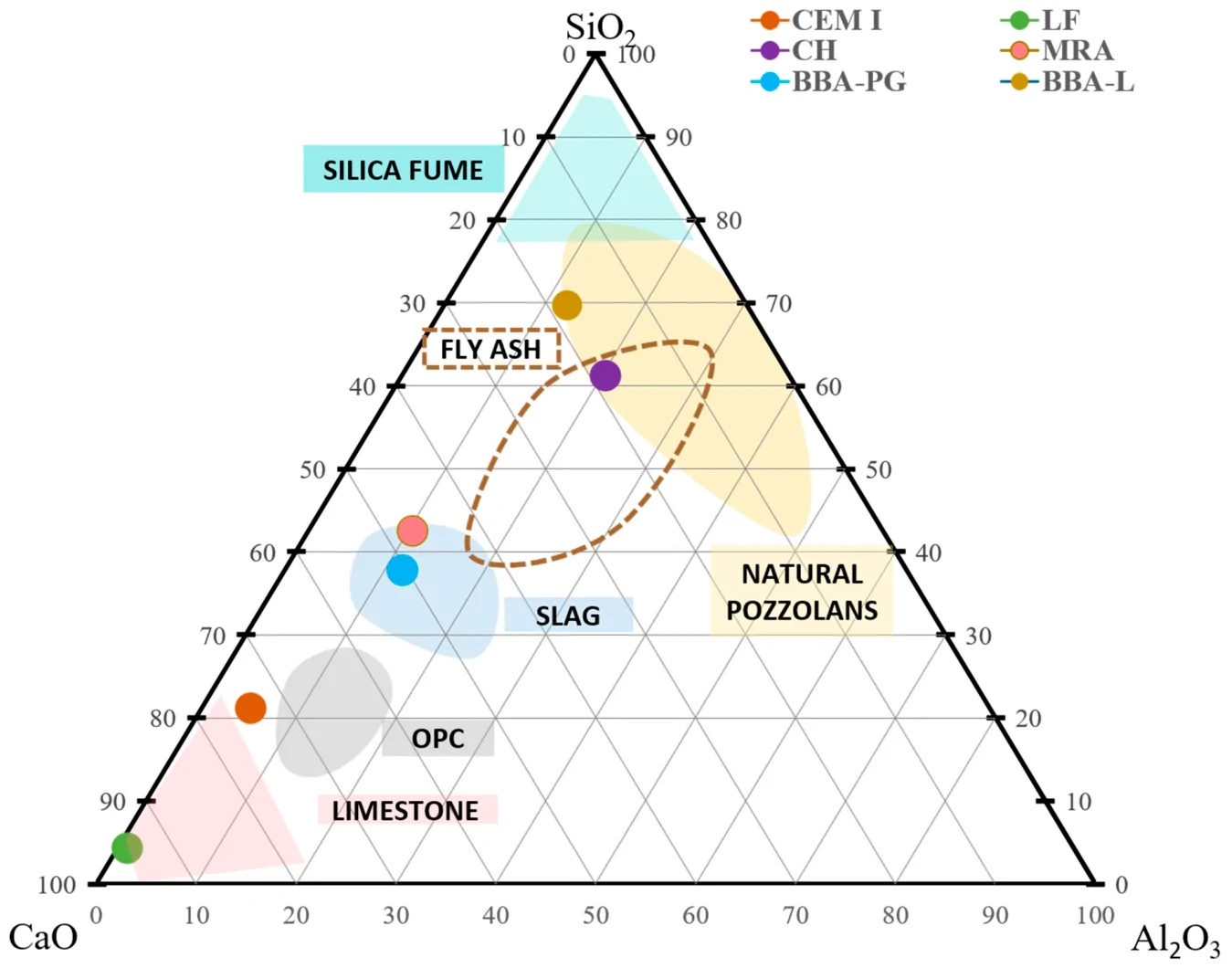
CaO-SiO_2_-Al_2_O_3_ ternary diagram of the supplementary cement materials analyzed.

The physico-chemical properties of each of the materials under study were analyzed. In addition, the materials were plotted on a ternary diagram to facilitate the selection of the most suitable materials for use as supplementary cementitious materials (Figure 4).

Figure 4 shows that the materials analyzed cover a wide range of chemical compositions. While CEM I, CH and LF are predominantly calcium-based materials, MRA, BBA-PG and BBA-L have higher silica contents and are closer to the range of pozzolanic materials. Among the latter, BBA-L has the highest silica content, followed by MRA, indicating greater potential for developing pozzolanic reactions and contributing to the formation of secondary cementitious products.

Once the materials had undergone a crushing process to be used as a partial cement substitute, an analysis of their physicochemical properties was conducted. Table 5 lists the main physical and chemical properties of the biomass ashes (pBBA-L and pBBA-PG), the powder derived from mixed recycled aggregates (pMRA), and chamotte (CH).

In general, the results show significant differences among the materials, both in terms of bulk density and Blaine fineness, which may influence their behavior during hydration and their effects on the properties of cementitious mixtures. The properties of each of the alternative materials analyzed are shown below.

#### 2.3.1. Pulverized Biomass Bottom Ash (pBBA)

**Figure 5:**
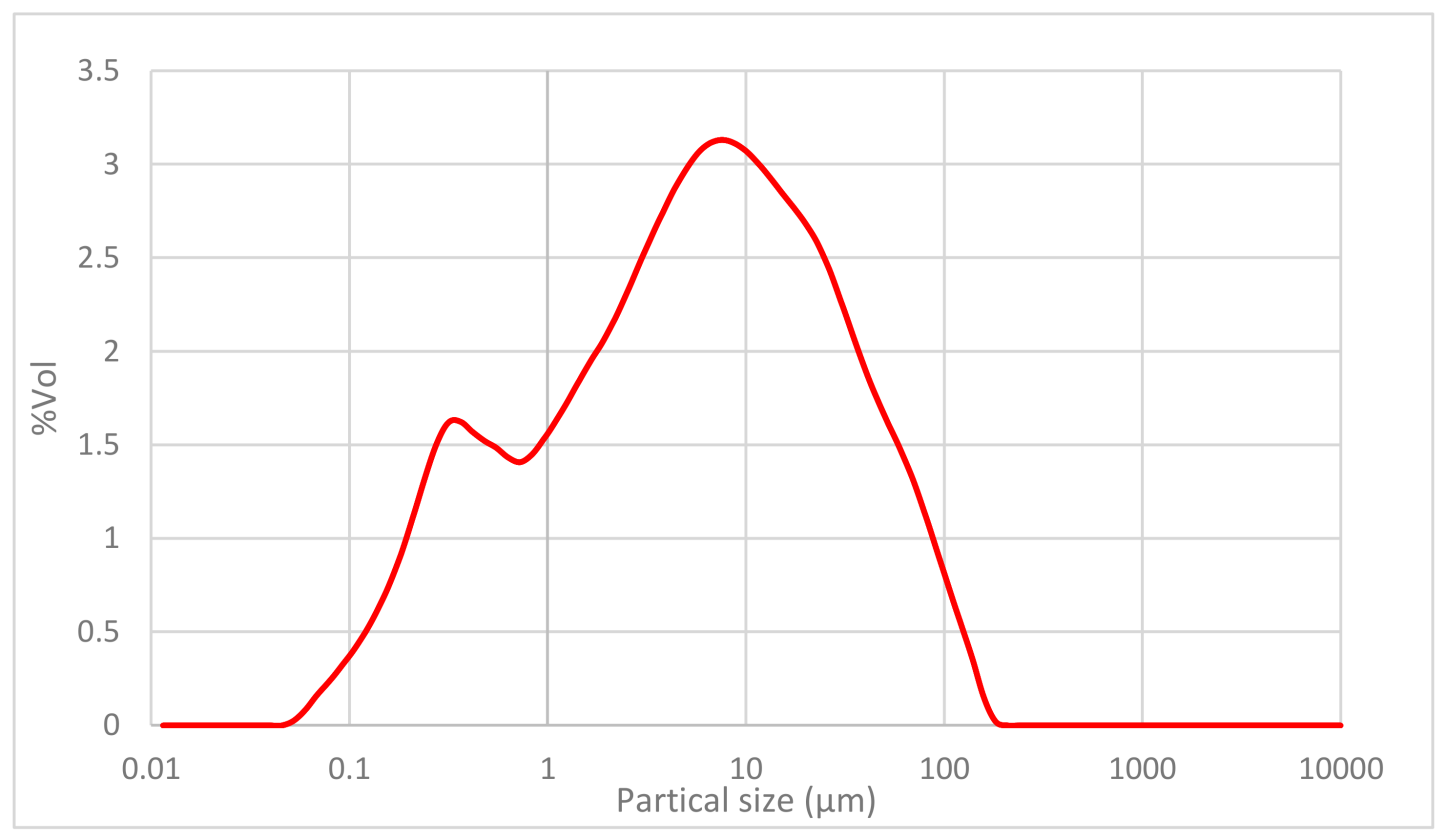
Particle size distribution of pBBA-PG (µm).

**Figure 6:**
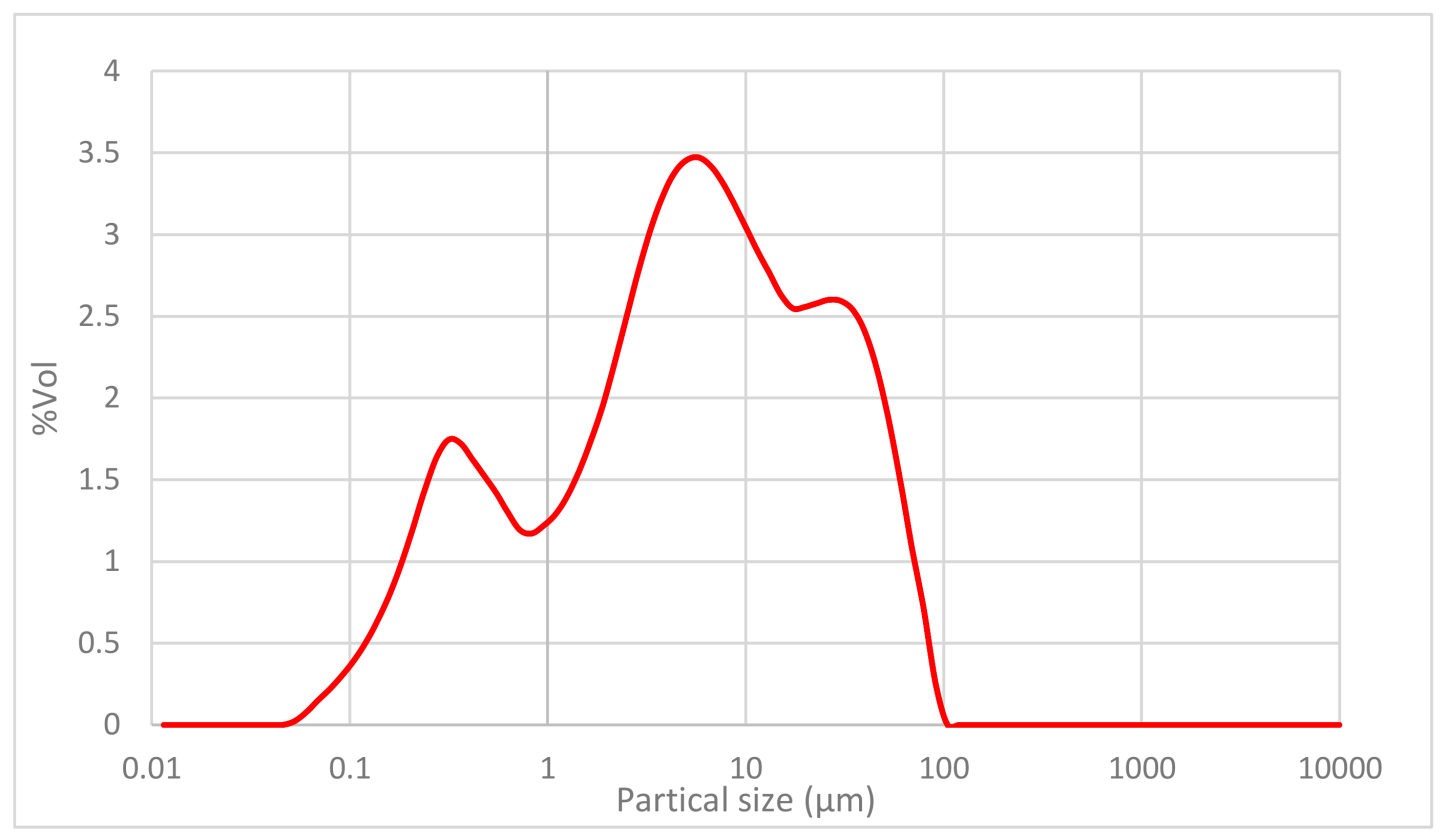
Particle size distribution of pBBA-L (µm).

As explained in Section 2, the biomass bottom ash, both from Linares and Puente Genil, was subjected to a crushing process. Firstly, both materials were crushed in a jaw crusher, then fed into a ring mill until the desired fineness was achieved. The particle size distribution of each of the materials following processing was analyzed using wet laser particle size analysis. The results are shown in Figure 5 and Figure 6.

According to the results shown in both figures, the particle size distribution is bimodal, combining a fine fraction centered around 0.3–0.5 µm and a second predominant fraction with particles ranging from approximately 5 to 10 µm.

From the perspective of material behavior, both distributions promote adequate particle packing by combining fine and medium fractions. Smaller particles can fill the voids between cement grains (filler effect), increasing the initial paste density and reducing capillary porosity. Furthermore, if the material possesses pozzolanic or latent hydraulic activity, as observed in Figure 4 for pBBA-L, the finer fraction provides a high specific surface area that promotes the dissolution of reactive phases and the additional formation of hydration products, primarily C-(A)-S-H gel, contributing to the refinement of the microstructure and the development of mechanical and durability properties.

Overall, the differences observed between the two curves are relatively small and suggest that both materials exhibit particle size characteristics compatible with their use as supplementary cementitious materials. However, the slightly more concentrated distribution shown in Figure 6 relative to pBBA-L could promote more efficient packing and greater uniformity of the hardened matrix, while the broader distribution shown in Figure 5 (pBBA-PG) could provide a better balance between the filling effect and the compaction of particles of different sizes.

As shown in Table 4, the real density of the pBBA samples was lower, mainly due to the more porous and heterogeneous nature typically found in these wastes.

With regard to Blaine fineness, pBBA-PG had the highest specific surface area (5733 cm^2^/g), followed by pBBA-L (5096 cm^2^/g). All materials therefore exhibit high fineness after grinding, exceeding that typically obtained for coarse fractions of these wastes. This characteristic is advantageous for their use as cement substitutes, since the increase in specific surface area expands the area available for interaction between the waste particles and the aqueous phase. In addition, the fine particles can exert a significant filling effect, occupying the interstitial spaces between the cement particles and contributing to a more refined pore structure. In potentially reactive materials, a finer particle size can also promote the rate of pozzolanic reaction [38].

The high Blaine fineness value indicates that this material may have a high capacity for interaction with cement paste. However, a high specific surface area can simultaneously increase the water demand of the mixture. According to previous studies, this effect is particularly significant in waste materials with irregular morphology or high porosity, in which the increase in available surface area leads to a reduction in workability [39].

In this regard, pBBA-L exhibited a composition particularly well-suited for a potential pozzolanic contribution, due to its high SiO_2_ concentration and combined pozzolanic oxide content exceeding 70%. Its high fineness (5096 cm^2^/g) may also enhance the interaction of the particles with the Ca(OH)_2_ generated during cement hydration. Biomass ashes generally exhibit significant potential as SCMs, although their performance depends heavily on the type of biomass, combustion conditions, and subsequent treatment of the ash [40].

pBBA-PG exhibited different values. Although its SiO_2_ content is lower than that of pBBA-L, it has a considerably higher CaO content (29.13%) and the highest Blaine fineness (5733 cm^2^/g). This combination could promote both a filler effect and participation in the hydration processes.

Its higher CaO/SiO_2_ ratio also suggests that its behavior may differ from that of a conventional siliceous pozzolan, potentially contributing in a more complex manner that combines hydraulic, pozzolanic, and physical mechanisms.

#### 2.3.2. Pulverized Mixed Recycled Aggregates (pMRA)

**Figure 7:**
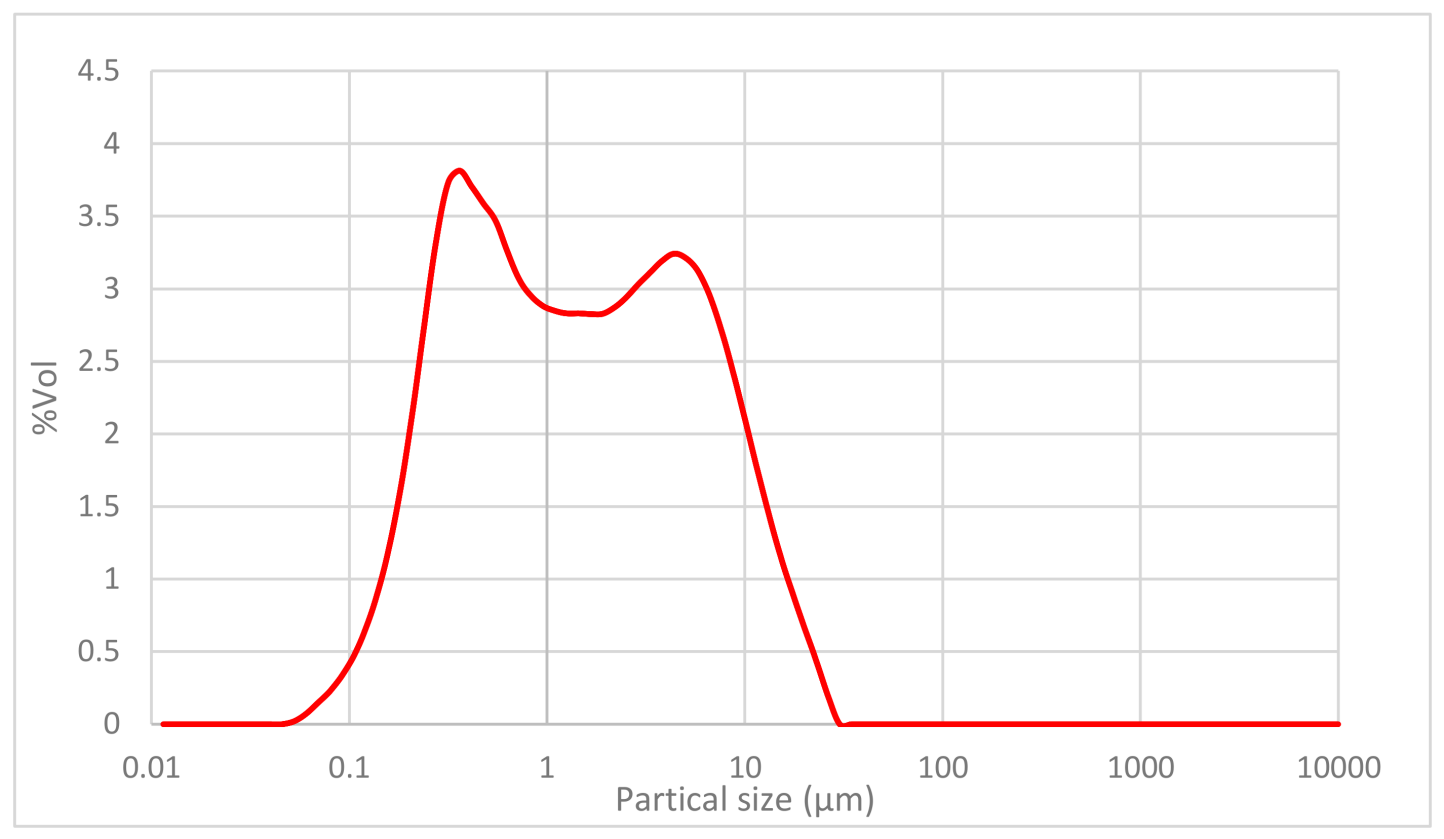
Particle size distribution of pMRA (µm).

As explained in Section 2, and in the same manner as was done with the other materials evaluated as cement substitutes, the mixed recycled aggregate underwent a crushing process. First, it was crushed in a jaw crusher and then fed into a ring mill until the desired fineness was achieved. The particle size distribution was analyzed using a wet laser method. The results are shown in Figure 7.

The particle size distribution of pMRA exhibited favorable characteristics, consistent with previous studies, for its use as a partial substitute for Portland cement following the processing it underwent. The presence of ultrafine particles can enhance the filling effect by occupying the spaces between cement grains and increasing the packing density of the cementitious matrix. As a result, capillary porosity is reduced, and the development of a more compact microstructure is promoted, which can lead to increased mechanical strength and improved durability of the hardened material [41].

Furthermore, as shown in the figure, a higher proportion of larger particles was observed; according to previous studies, this helps optimize the system’s particle size distribution, promoting the packing of solid particles and reducing water demand when moderate substitution percentages are used [38].

From a physical standpoint, the pMRA exhibited a higher bulk density (2520 kg/m^3^) due to its predominantly mineral origin and the presence of various components derived from construction and demolition waste.

The pMRA, with a Blaine surface area of 5512 cm^2^/g, exhibited high fineness but was lower than that of the other materials analyzed. Combined with its higher density, this can provide good compaction of the cementitious matrix. This result is particularly interesting because recent studies have shown that powders obtained through the processing of mixed recycled aggregates can act not only as filler particles but also contribute to cement hydration and exhibit some pozzolanic activity at later stages. In particular, it has been observed that MRA powders can promote hydration, favor the formation of AFm phases, and contribute to the refinement of the porous structure of cement pastes [42].

The chemical composition of pMRA shown in Table 5 indicates that the CaO content is 58.54%, a high value; however, the sum of SiO_2_ + Al_2_O_3_ + Fe_2_O_3_ is only approximately 23%. This indicates that pMRA does not have the chemical composition typical of a silico-aluminous pozzolan. However, the high CaO content may be related to the presence of residues of hydrated cement paste originating from the recycled aggregate, while the high fineness obtained after crushing may enhance the filler effect.

#### 2.3.3. Chamotte (pCH)

This material consists of ceramic waste from defective or broken fired clay pieces that have been crushed, supplied by Cerámica Malpesa SA Company, Jaén, Spain. This material underwent a crushing process described in Section 2.

**Figure 8:**
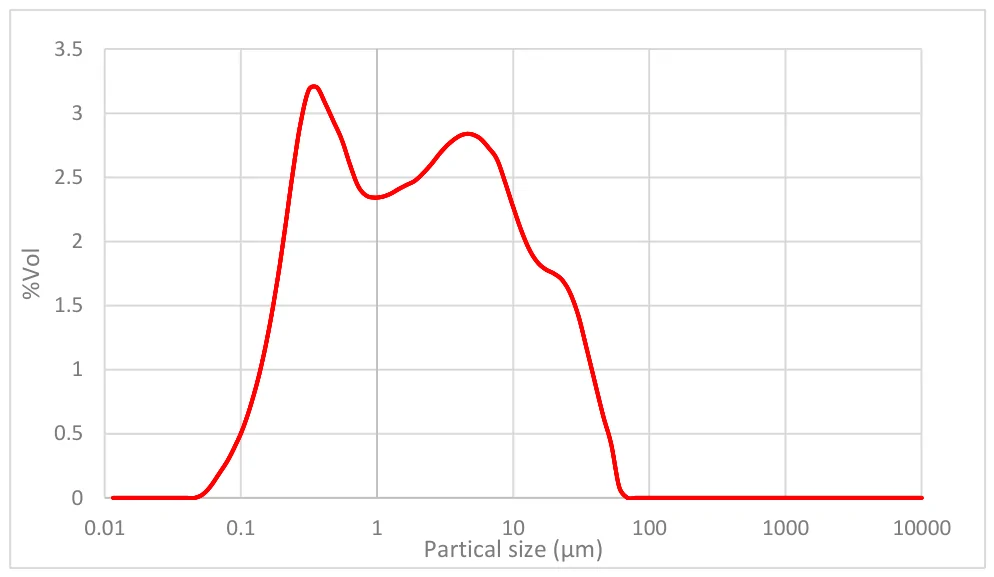
Particle size distribution of pCH (µm).

The curve shown in Figure 8 exhibited clearly bimodal behavior, as did the rest of the materials analyzed as partial cement substitutes.

The particle size distribution curve showed a relatively fine and broadly distributed material, with a significant proportion of particles smaller than 10 µm and a virtually total absence of particles larger than 70 µm. These characteristics are potentially favorable for its incorporation into cementitious systems, as they can simultaneously contribute to filling interparticle voids, increasing matrix densification, and, if reactive phases are present, the development of secondary hydration reactions.

From a physical standpoint, the results shown in Table 4 indicated that pCH had the lowest Blaine fineness value of the four materials (4756 cm^2^/g). Even so, this value remains high and is potentially suitable for use as a partial cement substitute. The aluminosilicate nature of ceramic waste means that its behavior can combine a physical filling effect with a pozzolanic contribution, particularly when the material has a high proportion of amorphous or reactive phases. In fact, it has been demonstrated that finely ground ceramic powders can participate in pozzolanic reactions with Ca(OH)_2_ and contribute to the refinement of the porous structure of cementitious materials [43].

Regarding chemical properties, pCH exhibited a high SiO_2_ content (49.90%) combined with an Al_2_O_3_ content of 17.60% and an Fe_2_O_3_ content of 6.81%, resulting in the highest combined content of potentially pozzolanic oxides, approximately 74.31%.

This composition is consistent with the aluminosilicate nature of ceramic and refractory waste. Furthermore, although CH has the lowest Blaine surface area of the materials studied (4756 cm^2^/g), its fineness remains high and may promote both the filler effect and the pozzolanic reaction [44].

## 3. Experimental Methods

In this study, three distinct series of mortars were prepared using different percentages and combinations of waste materials that had undergone different processing methods. First, mortars with a 25% partial replacement of cement with various processed byproducts; second, mortars with a partial replacement of sand; and third, a selection of optimal materials for producing mortars with a partial replacement of both cement and sand.

The experimental program evaluated the physical, mechanical, durability, and microstructural properties of the mortars produced. Table 6 shows the tests performed, the ages at which they were conducted, and the applicable standards.

### Mortar Mixture

The mortars were prepared following a standardized mechanical mixing protocol in accordance with the UNE-EN 196-1 standard. In the first stage, the cement or SCM used and the water were added to the mixer, and the mixture was mixed at low speed for 30 s. Next, the aggregates—composed of natural sand and the applied by-products—were added, along with pre-wetting water for each by-product to maintain a similar consistency, and the mixture was stirred slowly for another 30 s. Subsequently, the mixture was stirred at high speed for 30 s, followed by a 90 s pause to allow the mixture to settle. Finally, the mixing was completed with a final rapid mix lasting 60 s.

Upon completion of the mixing, the mortar was immediately poured into molds in two successive layers. Each layer was compacted by striking it 60 times on a vibrating table to ensure good densification and homogeneity of the material. The molds were stored in a humidity chamber at a constant temperature of 20 ± 2 °C and humidity of 95 ± 5 for 24 h, after which the specimens were demoulded. The demoulded specimens were kept in a chamber at a temperature and humidity of 20 ± 2 °C and 95 ± 5, respectively (until the time of testing.

Table 7 below shows three distinct series. Series 0 includes a control mortar made from 100% CEM I and a reference mortar consisting of 75% CEM I and 25% LF.

Series 1 shows mortars produced with 25% cement substitution. Series 2 shows mortars in which sand was replaced with various byproducts, and Series 3 shows a combination of cement and sand substitution. The aggregate substitution was performed by volume, due to the difference in density between NS and the other by-products being evaluated. To perform the volume substitutions, the density values for each aggregate listed in Table 2 were used. A density coefficient was applied to each substitution, calculated as the density of natural sand divided by the density of the recycled replacement material.

To maintain the workability of the mortars, the water-to-cement ratio was adjusted, taking into account the water absorption of the recycled material used as a sand substitute. The effective water-to-cement ratio increased from 0.5 in mixtures without recycled material to 0.6 in mixtures made with recycled material having a higher absorption rate.

It was decided to maintain workability at a constant level between 17 and 16 cm.

It should be noted that, since this study evaluates the thermal properties of the mortars, expanded clay (EC) was added to reduce the density of the mortars and improve their thermal insulation properties.

## 4. Results and Discussion

The results obtained for each of the properties evaluated in the designed mortars are shown below.

### 4.1. Mechanical Performance: Compressive and Flexural Strength

**Figure 9:**
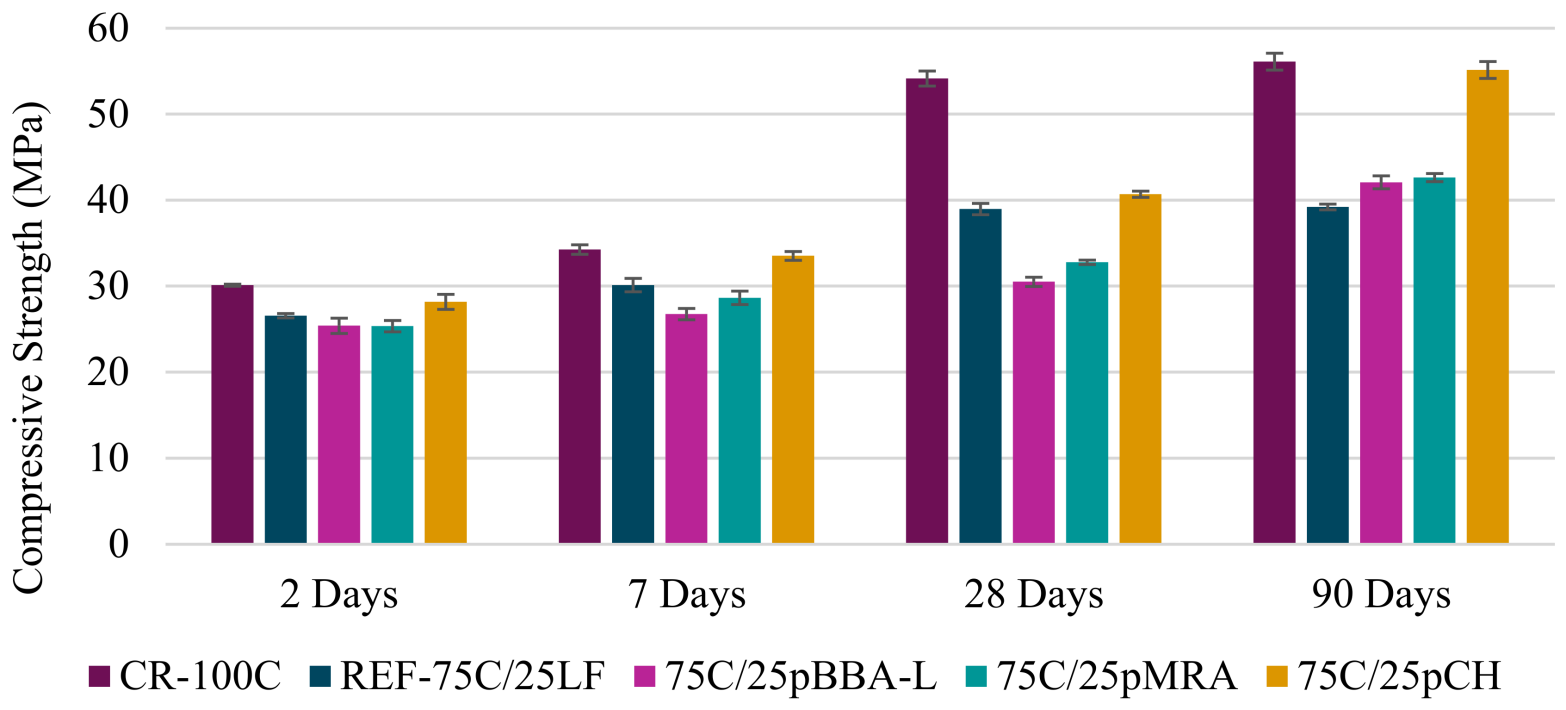
Compressive strength for mortars with cement replacement.

The compressive strength test was conducted at 2, 7, 28, and 90 days to illustrate the behavior of the mortar’s strength over time (Figure 9). As shown in the figure, it is evident that the compressive strength of all samples increased steadily with curing time due to ongoing hydration [13,48]. To evaluate the material as a partial substitute for cement, a study was conducted on the mechanical properties of two mortars used as controls: Control Mortar (CR-100C) and a reference mortar made with 25% limestone filler (REF-75C/25LF).

The latter mortar, the reference mortar, was selected as the reference for comparing the other mixtures and their properties.

It was observed that all mixtures had a lower compressive strength at 28 days compared to the control (CR). The reference series decreased by 28% at 28 days and by 30% at 90 days. However, the long-term use of pCH led to an improvement in compressive strength, with a reduction of 25% at 28 days and only 4% at 90 days compared to the control (CR-100C).

The reference mix 75C/25LF, which contains 25% limestone filler, showed a relative increase between 2 and 90 days of approximately 50%, reaching a value of 38.96 MPa at 28 days. The behavior of the limestone filler can be attributed primarily to a physical filling effect and improved particle packing, rather than to a pozzolanic contribution comparable to that of other aluminosilicate materials. Recent literature confirms that the effect of the limestone filler depends considerably on its proportion, fineness, water-to-binder ratio, and particle size distribution. Dos Santos et al. (2024) [49] observed that the partial replacement of cement with limestone filler can yield satisfactory mechanical results when particle packing is optimized, while Jiang et al. (2024) [50] noted that high filler contents can lead to a reduction in strength, especially as the water-to-binder ratio increases.

In the case of 75C/25pBBA-L, which incorporated 25% biomass bottom ash as a cement substitute, the initial strength was slightly lower than that of the control (25.4 MPa versus 26.6 MPa at 2 days and 26.7 MPa versus 30.1 MPa at 7 days). At 28 days, the strength reached 30.5 MPa, still below that of the reference mortar; however, at 90 days, a significant change occurred, reaching 42.1 MPa, approximately 8% higher than the control’s strength. This behavior suggests a progressive contribution of the biomass bottom ash at advanced ages. The lower initial strength may be related to the so-called cement dilution effect, as well as to the high porosity, surface irregularity, and water demand that certain bottom ash types may exhibit. However, when the ash is properly processed through grinding, its reactivity can be significantly increased. Rosales et al. (2024) [51] specifically studied substitutions of 25% of the cement with pulverized biomass bottom ash, obtaining 28-day strengths comparable to and even higher than those of the reference cement, depending on the origin of the ash. Likewise, recent studies highlight that the chemical composition and pretreatment of the ash are determining factors for its mechanical behavior [18].

Similar behavior at advanced ages was observed in the 75C/25pMRA mixture. At 28 days, the strength is approximately 15% lower than that of the control; however, at 90 days, it reaches a value about 10% higher than that of the REF-75C/25LF mixture. This behavior is particularly interesting because it indicates that the use of recycled material does not necessarily imply a permanent loss of mechanical properties. In this regard, Liu et al. (2025) [42] demonstrated that powders derived from mixed recycled aggregates can act as a supplementary cementitious material and that, with a 25% cement replacement, they can contribute favorably to strength development, especially at advanced ages.

Other authors, such as Agrela et al. (2024) [52], demonstrated that the combination of mixed recycled aggregate and biomass bottom ash moderately reduced mechanical strength but yielded environmental benefits resulting from reduced consumption of cement and natural raw materials.

The most favorable mechanical behavior of all the mixtures was observed in the 75C/25pCH mixture [53]; at 90 days, it exceeded the control by approximately 16 MPa, equivalent to a 41% increase. Furthermore, as early as 28 days, it exhibited slightly higher strength than the control, demonstrating that the beneficial effect of chamotte is evident at both intermediate and long ages. This behavior may be related to the silico-aluminous nature of the chamotte, as shown in Table 4; its high thermal stability and fine particle size result in a combined effect of filling, improved packing, and reactivity of the aluminosilicate phases. García-Díaz et al. (2024) [53] studied the incorporation of chamotte derived from ceramic waste into alkali-activated cements and observed that its addition significantly modified the mechanical and microstructural properties of the system, highlighting the potential of this waste as a component of sustainable cementitious materials. Similarly, Sinha et al. (2023) [54] analyzed concrete containing refractory brick waste and confirmed the potential of this waste to produce sustainable cementitious materials with adequate mechanical performance.

**Figure 10:**
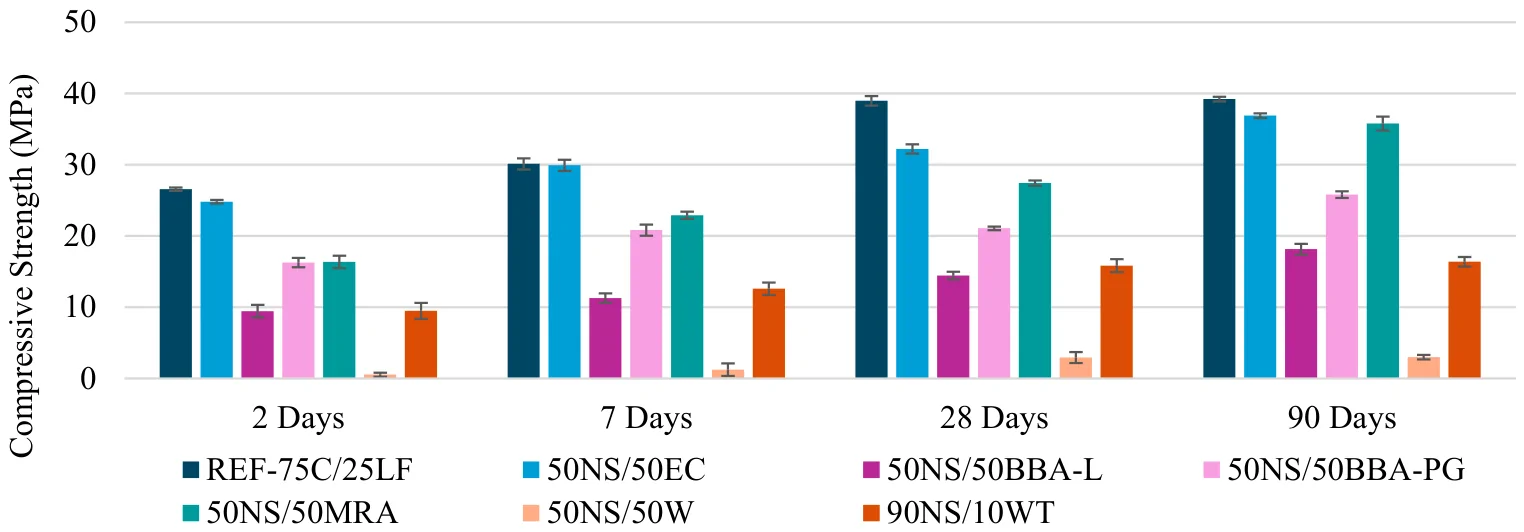
Compressive strength for mortars with sand replacement.

In addition to the partial replacement of cement, the replacement of a high proportion of natural sand with various byproducts was evaluated. Figure 10 shows the compressive strength results obtained.

As shown in the figure, replacing natural sand with waste materials does not have a uniform effect on compressive strength; rather, it depends on the nature of the substitute and its interaction with the cementitious matrix. Mineral or ceramic materials with greater stability, such as EC and MRA, exhibited a smaller loss of strength compared to highly porous or organic materials (BBA and W).

The use of EC as a partial substitute for sand in mortars to reduce density and improve thermal properties resulted in an 18% reduction in 28-day compressive strength compared to CR; however, at 90 days, this loss was reduced to 5%. The behavior of 50NS/50EC can be explained primarily by the characteristics of expanded clay. This material has a highly porous internal structure. The porosity of expanded clay is precisely one of the main factors that determine the mechanical properties of lightweight concrete and mortar [55,56].

The addition of MRA to mortars resulted in a noticeable reduction in compressive strength at early ages; however, the difference decreases significantly with curing and reaches only 9% at 90 days.

This evolution in strength indicates that the MRA-containing matrix continues to develop strength significantly during the extended curing period. The explanation may be related to the presence of adhered mortar particles and ceramic fractions in the mixed recycled aggregate. These particles exhibit greater roughness and water absorption than natural sand and can modify water availability during hydration, in addition to promoting a certain degree of mechanical interaction with the paste.

The results are consistent with research by other authors. Agrela et al. (2024) [52] studied cementitious systems incorporating MRA and BBA and noted that the physical characteristics of MRA, particularly its density, water absorption, and composition, strongly influence the material’s behavior.

Unlike the application of MRA, the use of BBA reduced the compressive strength of the mortars analyzed, showing a marked dependence on its origin. The approximately 42% difference in 90-day compressive strength values between the application of BBA-PG and BBA-L demonstrates that the origin of the ash should be considered a fundamental variable in mix design.

The 50NS/50BBA-PG mixture exhibited clearly superior performance compared to the 50NS/50BBA-L mixture. The difference in performance between the two biomass ash types can be explained by their physical properties, as shown in Table 2. BBA-PG showed a density of 2.12 g/cm^3^ and a water absorption of 11.9%, while BBA-L presented a considerably lower density of 1.89 g/cm^3^ and a higher absorption of 23.84%. These differences are relevant because the density and absorption of BBAs are closely related to their porosity and the amount of water they can retain during mixing and curing [33].

The higher absorption of BBA-L can have a particularly unfavorable influence on compressive strength. When using a 50% sand replacement, a significant portion of the mixing water may initially be retained in the pores of the BBA particles, even after saturation water has been added to the mix. This reduces the amount of free water available for cement hydration and simultaneously hinders the workability of the mix. This effect can lead to a less homogeneous paste distribution and promote the formation of a more porous structure. Conversely, the lower absorption of BBA-PG implies a lower water demand from the aggregate and, predictably, less alteration of the effective water/cement ratio.

The use of another porous, low-density waste product, such as wood by-products, as a partial substitute for aggregate in mortar manufacturing resulted in significantly reduced compressive strengths, reaching 92% lower than the control at 90 days. This behavior can be attributed to a combination of factors related to the lignocellulosic nature of the waste. Wood has considerably lower density and stiffness than mineral aggregates, as well as a high water absorption capacity (Table 2). Furthermore, the organic compounds present in wood, such as lignin, hemicelluloses, and extractives, can interfere with cement hydration and the formation of a suitable transition zone between the particle and the cementitious matrix.

Alrubaie et al. (2023) [57] observed that incorporating wood waste as a substitute for conventional aggregates can lead to significant reductions in compressive strength due to the lower stiffness and high absorption of the particles. The authors also demonstrated that pretreating the wood can significantly improve the interaction between the residue and the cementitious matrix. For this reason, the study aimed to evaluate the wood under alkaline treatment, and considering the results obtained, a reduction in the percentage of additive was considered. The strength shown by the 90NS/10WT mortar is approximately 5.2 times greater than that of the 50NS/50WT mortar, representing an increase of nearly 416%.

According to previous studies, alkaline treatment can modify the surface of lignocellulosic particles by partially removing non-cellulosic components and by changing the surface roughness and wettability. These modifications can improve the adhesion between the wood and the cement paste and reduce some of the negative effects associated with the presence of organic compounds.

**Figure 11:**
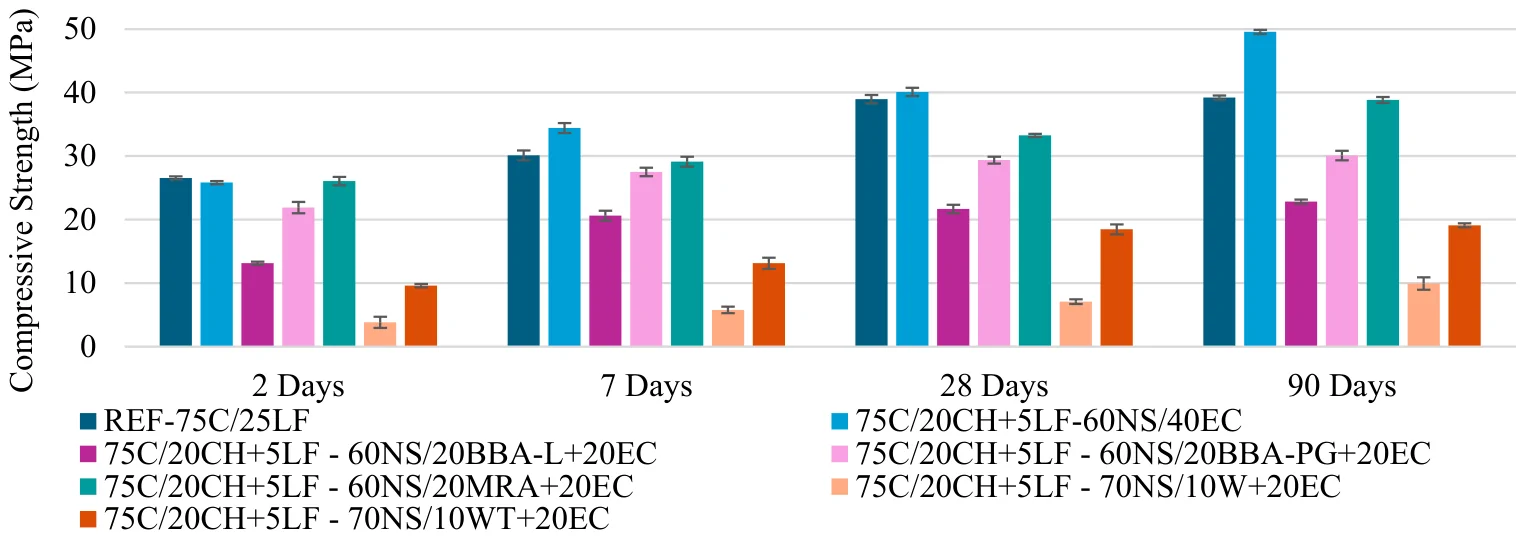
Compressive Strength for mortars with cement and sand replacement.

Based on the results obtained in the evaluation of partial cement and aggregate substitution, a combination of solutions was implemented to reduce the amount of cement and introduce recycled material as aggregate, seeking suitable mechanical properties and thermal insulation. All mixtures were determined to use 75% cement, 20% chamotte, and 5% limestone filler. As aggregate substitutes, a combination of 20% expanded clay with 20% of the different byproducts was evaluated. The results obtained are shown in Figure 11.

The 75C/20pCH+5LF-60NS/40EC mixture exhibited the most favorable mechanical behavior of all the formulations, reaching 49.6 MPa at 90 days. This behavior is particularly interesting because expanded clay, due to its high porosity, typically tends to reduce mechanical strength. Uysal et al. (2024) [56] noted that the porosity and absorption of expanded clay can lead to a decrease in strength compared to conventional aggregates. However, in this case, this effect appears to be offset by the modification of the cementitious phase through the incorporation of chamotte.

One possible explanation is that the chamotte acts simultaneously through a filler effect and a pozzolanic reaction, while the expanded clay provides a porous internal surface capable of modifying water distribution and promoting some internal curing.

The incorporation of BBA resulted in clearly different behavior, considerably reducing strength compared to the control. The differences in density and absorption between the two BBAs analyzed (Table 2) may be the factors that substantially modify water availability during mixing and hydration, especially when combined with an additional 20% of expanded clay. For this reason, BBA-L, with greater porosity, produced a reduction in strength compared to BBA-PG.

The figure shows that the combined incorporation of chamotte, limestone filler, MRA (medium-density aggregate), and expanded clay achieves mechanical strength equivalent to the control material in the long term, despite the inclusion of a considerable amount of recycled materials. This behavior may be related to the high surface roughness of the MRA and its ability to establish good mechanical interaction with the paste. The presence of adhered mortar and ceramic particles can increase absorption but also promote good adhesion in the interfacial transition zone. Recent research has shown that the combined use of recycled materials and mineral waste can maintain or even improve mechanical properties [57].

Formulations incorporating wood waste exhibit the lowest strengths, although a very clear difference is observed between untreated and treated wood. The simultaneous presence of 20% expanded clay and 10% wood generates a granular skeleton with a high proportion of porous particles and low stiffness. Treatment with NaOH can partially remove non-cellulosic and extractive components from the particle surface, modifying their roughness, wettability, and adhesion to the cement paste. This can improve the interfacial transition zone and reduce some of the negative effects of wood on hydration, as can be seen in the compressive strength results obtained.

Table 8 shows the 90-day compressive strength results for all mixtures compared to the reference mortar. These relative results allow for a clear classification of the substitution strategies. The use of chamotte as a partial cement substitute led to a 40.96% increase in strength compared to the control.

In the evaluation of lightweight mortars using expanded clay, the combination with chamotte was the only formulation that provided a significant improvement over the control (+25.6%), while the incorporation of MRA as a partial aggregate substitute practically maintained the strength of the control mortar. BBA produced an intermediate effect, but one strongly influenced by its origin: BBA-PG was considerably more favorable than BBA-L. Finally, wood waste resulted in the greatest reduction, although treatment with NaOH substantially reduced this loss.

Table 9 shows the evolution of the flexural strength of the mortars at 2, 7, 28, and 90 days. In general, all formulations showed an increase in strength with curing age, as did the compressive strength (Figure 9, Figure 10 and Figure 11), although the magnitude of this evolution depended significantly on the nature of the material used as a substitute and whether the substitution affected the cement, the aggregate fraction, or both.

Replacing cement with different byproducts resulted in an improvement over the control mortar made with limestone filler. It is worth noting that the cement substitutions corresponded to 75C/25pCH. At 28 days, it outperformed the control by 16.9%. This result may be related to the ceramic nature of chamotte and its potential pozzolanic activity, as observed in Figure 4. Finely ground ceramic waste can provide reactive silica and alumina and simultaneously act as a filler material, promoting matrix densification and the development of secondary hydration products. Recent studies have shown that ceramic waste can be used as a cement substitute, maintaining or even improving flexural strength, especially at medium and long ages [58]. Furthermore, it has been observed that certain ceramic demolition waste exhibits sufficient pozzolanic activity to replace up to approximately 20% of the cement without a significant penalty to 90-day performance [59].

Greater differences were observed when alternative materials replaced natural sand.

The incorporation of EC introduced a light, porous granular fraction that can increase water absorption and internal porosity, reducing flexural strength. The performance of BBA as a sand substitute was much less favorable. The difference between BBA-L and BBA-PG is especially relevant given the previously determined physical properties of both ash types. BBA-L exhibited higher absorption than BBA-PG. This high absorption can modify water availability in the mixture and favor a more porous structure, which is particularly detrimental to flexural strength due to the sensitivity of this property to defects, pores, and discontinuities in the matrix [60].

The 50NS/50MRA mix showed considerably better performance. The roughness and texture of the recycled particles may contribute to a more homogeneous internal distribution within the paste, although the presence of adhered mortar and its greater absorption may explain why the strength does not exceed the values of the reference mortar.

The most unfavorable results were obtained with the formulations containing wood waste (W and WT). This behavior can be explained by the high porosity and hydrophilic nature of the lignocellulosic particles, as well as their low stiffness and potential interference with cement hydration. The alkaline treatment produced a significant improvement, as was also observed with compressive strength. Previous studies have shown that the alkaline treatment of plant fibers improves adhesion to the matrix and can increase flexural strength and favorably modify the fracture mechanism [61].

Analyzing the material combination as a partial substitute for cement and aggregate, the use of BBA-PG and MRA led to similar results to those of the control mortar.

### 4.2. Physical and Durability Properties

The following details each of the physical and durability properties analyzed to evaluate mortars as a material with thermal insulation characteristics.

#### 4.2.1. Bulk Density and Porosity

Table 10 presents the bulk density and water accessible porosity values of the mortars after 28 days of curing.

We measured the water-accessible porosity of the hardened mortar specimens using the method in UNE 83980:2014 [62], with adjustments for mortar. First, we dried the specimens, then saturated them with water using the boiling method. We recorded the dry mass (m_1_), the mass after boiling or vacuum (m_3_), and the apparent (hydrostatic) mass (m_4_). We then calculated the water-accessible porosity using the equation:

(1) $$\mathrm{accecible}\;\mathrm{porosity}=\frac{m3-m1}{m3-m4}\times 100$$

where (m_1_) is the dry mass of the specimen, (m_3_) is the mass after boiling, and (m_4_) is the apparent (hydrostatic) mass of the specimen.

In general, the results showed an inverse relationship between density and porosity, particularly marked in mortars that incorporated lightweight or highly porous materials. This trend is consistent with recent studies, which indicate that mortar density is closely related to pore volume and the physical nature of the substitute materials [56].

The results showed three clearly differentiated behaviors in each of the analyzed series. First, cement substitutions barely modified the physical structure of the mortar: density variations were less than 2.5% and porosity variations less than 1.5%.

Secondly, the sand substitutions resulted in much more significant changes, especially when highly porous materials such as BBA-L and W were used. The increase in porosity observed for MRA is much smaller than that for BBA-L or wood. This helps explain why, in the previously obtained mechanical results, 50NS/50MRA exhibited a 90-day flexural strength of approximately 7.81 MPa, very close to the control (Table 9). Therefore, MRA demonstrated a favorable balance between density reduction and preservation of mechanical performance.

**Figure 12:**
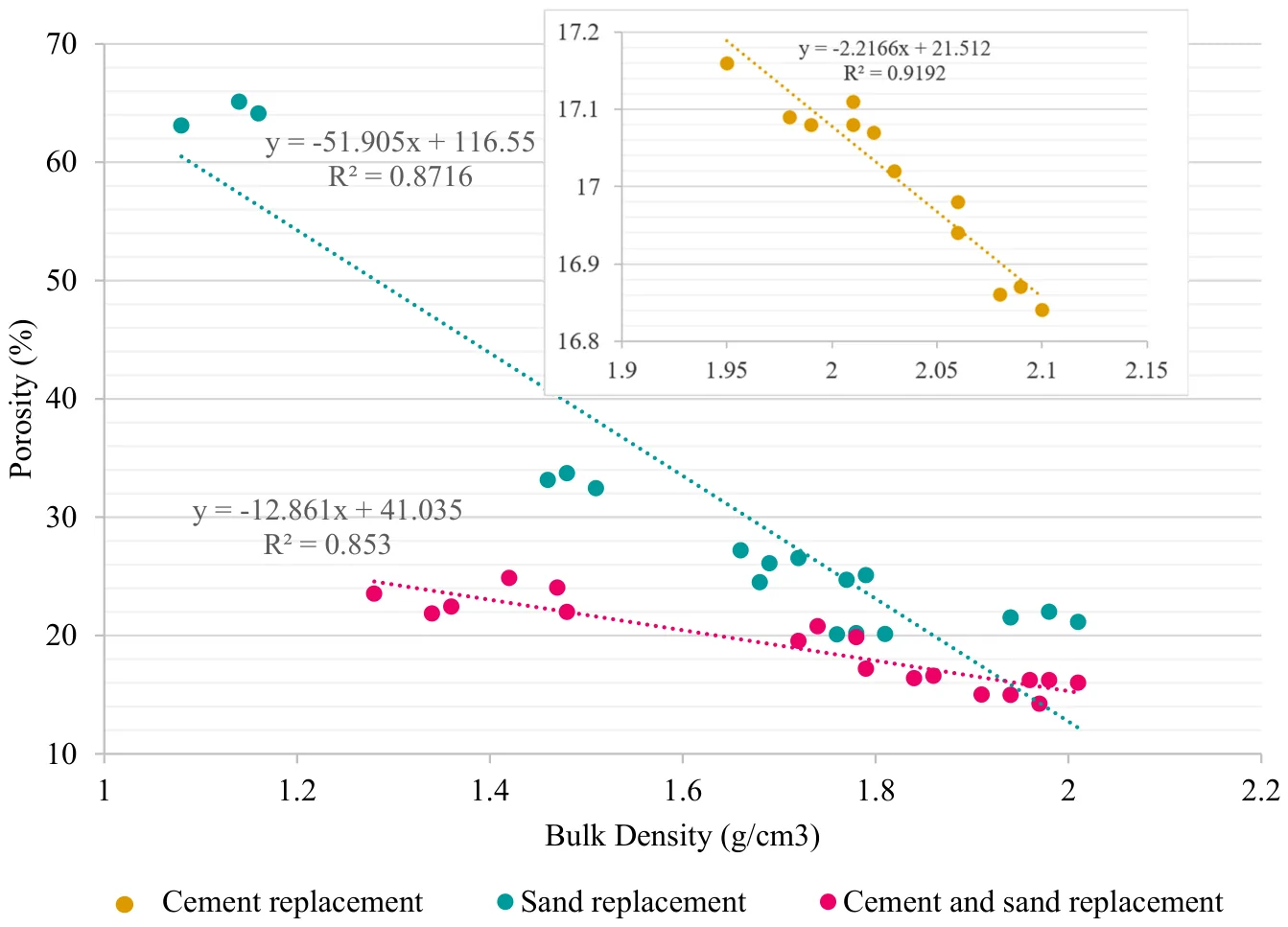
Relationship between bulk density and porosity of mortars performed by series.

The relationship between bulk density and porosity of the mortars was further analyzed in Figure 12.

Overall, a negative correlation was observed between both properties, such that the increase in density is associated with a reduction in porosity. The strength of this relationship depended on the type of substitution. For Series 1, a very high linear correlation was observed, although the slope was relatively small, indicating that relatively minor modifications to the porous structure occurred. For the mixtures in which the natural sand was replaced (Series 2), considering the slope of the line, it was observed that the substitution of the granular fraction had a much more pronounced impact on the porous structure of the mortar than in Series 1. In Series 3, it was observed that the combination of both substitutions generated a less pronounced porosity response to density than that observed solely for the sand substitution. This result indicated that modifying the cementitious phase with chamotte and limestone filler can partially compensate for the effect of lightweight or porous aggregates, promoting matrix packing [63].

The observed correlation proved particularly important when relating these physical properties to the previously determined mechanical strengths. Generally speaking, mixtures with higher density and lower porosity exhibited better mechanical performance, while increased porosity resulted in reduced strength. This relationship has also been observed in mortars containing BBA and construction and demolition waste, where a correlation between porosity and mechanical strength has been found [5].

Overall, the high correlation observed between apparent density and porosity confirmed that the modification of the porous structure is one of the main mechanisms by which the incorporated residues condition the physical and mechanical performance of mortars; however, the differences in the slopes of the regressions of each of the analyzed series show that the nature and function of the residue (as a substitute for cement or sand) are determining factors in the final response of the material [64,65].

#### 4.2.2. Ultrasonic Pulse Velocity (UPV)

Table 11 presents the ultrasonic pulse velocity (UPV) values determined at 2, 7, 28, and 90 days for the different mortar formulations. In general, the results showed an increase in ultrasonic velocity with curing age for virtually all mixtures, indicating a progressive densification and consolidation of the cementitious matrix. This behavior is related to the evolution of hydration reactions and the progressive formation of hydration products that reduce the number and connectivity of pores. Cantero et al. (2022) [66] also observed an increase in UPV with curing age in mortars with supplementary cementitious materials, attributing it to the progressive filling of internal pores by hydration products.

The reference mortar REF-75C/25LF showed an increase of approximately 15.1% between 2 and 90 days, confirming the normal evolution of the cementitious matrix. Values close to 4.4 km/s at 90 days indicate a relatively compact structure. The compressive strength (CS) is, in fact, an indirect indicator of the material’s quality and internal continuity, being conditioned by its density, porosity, composition, and aggregate-paste interface characteristics. Zhang et al. (2024) [67] found a significant correlation between PS, density, and other mechanical properties of cementitious materials, confirming the usefulness of this technique as a non-destructive method.

The partial replacement of cement with each of the analyzed SCMs did not significantly influence the UPV values, with a correlation coefficient between the two variables of R^2^ = 0.64. The most notable performance was observed with 75C/25pCH. Although at 2 days it presented a slightly lower UPV than the control (3725 m/s; −2.03%), from 7 days onwards it clearly exceeded the reference, reaching 4487 m/s at 28 days (+5.23%) and 4536 m/s at 90 days (+3.61%). These results show that the application of chamotte as a cement substitute improves progressively with age, possibly due to a combination of filler effect, improved packing, and pozzolanic reactivity, which favors the formation of a more continuous matrix.

This result is consistent with recent research on ceramic waste, which has shown that the fineness and composition of waste influence its ability to modify the microstructure of cementitious materials. Consequently, the behavior of 75C/25pCH suggests that replacing cement with chamotte not only maintains internal continuity but can also contribute to improving it at advanced ages.

Greater changes were observed when the substitution was made on natural sand, confirming what had been previously observed for density and porosity. Shahniani et al. (2025) [68] specifically observed that increasing the content of lightweight expanded clay reduces density and negatively modifies the mechanical properties and UPV of cementitious materials.

The application of BBA resulted in a greater variation in UPV values. The high porosity of BBA-L generated greater dispersion and attenuation of the ultrasonic waves, which was somewhat reduced with the use of BBA-PG due to its lower porosity. Specific literature on BBA confirms that its high surface porosity and absorption capacity can negatively affect ultrasonic propagation when used as a substitute for aggregates [33].

In the case of MRA application, a different result was observed. Despite the high sand replacement, the matrix continued to progressively develop its internal structure, as shown by the lower UPV result compared to the control. This value can be attributed to the greater porosity of the MRA and, especially, to the presence of mortar adhered to the recycled particles, which introduces an additional interface and areas with varying stiffness.

The application of wood waste showed that, according to UPV values, a highly discontinuous internal structure was generated. Wood has a cellular structure and a much lower density than mineral aggregates, in addition to a high absorption capacity. These characteristics hinder wave transmission and generate multiple wood-paste interfaces.

The hybrid mixtures (Series 3) showed intermediate performance, although some formulations exhibited a particularly favorable response. In particular, the incorporation of chamotte and limestone filler in the cementitious phase, combined with EC or MRA, allowed for the maintenance of relatively high UPV values.

**Figure 13:**
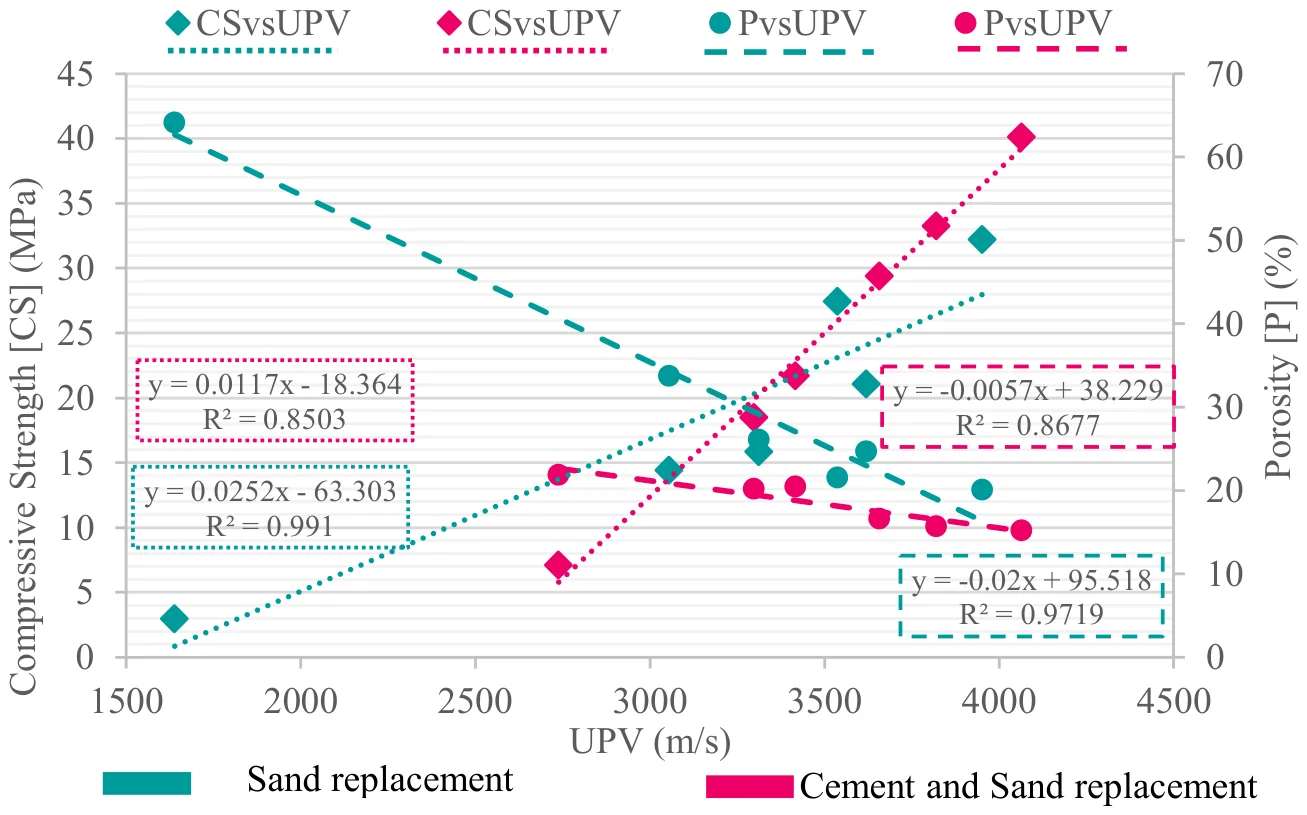
Correlation between ultrasonic pulse velocity (UPV), compressive strength (CS), and porosity (P) of mortars with different cement and aggregate replacement.

An analysis was conducted jointly of the relationship between ultrasonic pulse velocity (UPV), compressive strength (CS), and porosity (P) of mortars, differentiating between mixtures with sand replacement and those combining cement and sand replacement (Figure 13). The results showed clearly defined trends and, in general, confirmed that UPV is a good indirect indicator of the quality of the hardened matrix.

In both series, the increase in ultrasonic velocity (UPV) was associated with an increase in compressive strength and a reduction in porosity. Particularly high correlations were obtained in the mixtures with sand replacement, with R^2^ = 0.991 for the CS–UPV ratio and R^2^ = 0.9719 for UPV–P. These results indicate that most of the variability in mechanical and physical properties can be explained by the evolution of the ultrasonic velocity. Conversely, the mixtures with simultaneous cement and sand replacement showed R^2^ values of 0.8503 and 0.8677 for the CS–UPV and P–UPV ratios, respectively, demonstrating a greater complexity in the material’s response because of the simultaneous modification of the cementitious phase and the granular skeleton. Overall, the results confirmed that greater continuity and densification of the matrix simultaneously favor ultrasonic wave propagation and mechanical strength, while increased porosity has the opposite effect.

#### 4.2.3. Thermal Properties

The thermal properties of mortars have been measured in accordance with ISO 8301 [69] and ASTM C518 requirements. The thermal conductivity of mortar depends on its structure, porosity, and density [70]. The sample to be tested is 40 × 40 × 3.5 cm at a curing age of 28 days. Prior to the test, they were oven-dried until reaching a constant mass. A heat flow meter instrument was used to measure the thermal conductivity through vertical and upward heat flow from the lower plate to the upper plate of the instrument. Each sample was exposed to different temperatures with a range of 2 degrees. The first is between 14 and 16 °C, the second between 29 and 31 °C, and the third between 39 and 41 °C.

Fourier’s law describes heat transfer in isotropic materials and is related to the area and thickness of the material. In normal conditions, heat flows against the temperature gradient. The following equation shows the heat transfer across the area and thickness [71].

(2) $$\frac{Q}{\Delta t}=\frac{\lambda .A}{d}.(T1-T2)$$

Table 12 shows the thermal conductivity of the mortars tested at three temperature ranges, as well as the average value for each formulation and its relative variation compared to the reference mortar REF-75C/25LF.

In general, all mixtures containing waste materials showed a reduction in thermal conductivity compared to the control, demonstrating the potential of these wastes to improve the thermal insulation properties of mortars. This trend is particularly pronounced when waste materials replace natural sand, due to the reduced density and increased pore volume associated with many of these materials. The relationship between density, porosity, and thermal conductivity has been widely observed in lightweight and recycled cementitious materials [72].

The reference mortar REF-75C/25LF exhibited conductivity representative of a relatively compact matrix, consistent with the previously determined density and porosity values. The increase in conductivity between the 14–16 °C range and the 39–41 °C range was approximately 1.32%, indicating that, within the analyzed temperature range, temperature has a relatively minor effect compared to the effect produced by the mortar’s composition and internal structure.

These results show that cement replacement produces a relatively limited thermal improvement compared to sand replacement. This is reasonable, since modifying the cementitious phase by 25% does not alter the granular skeleton or the total pore volume as markedly as when 50% of the natural sand is replaced. However, the progressive reduction observed first with BBA-L, followed by MRA, and a greater reduction with CH indicates that the nature of the cementitious substitute also influences heat transfer.

The lower thermal conductivity of the mixture containing chamotte may be related to its ceramic nature and to the combined effect of its physical characteristics, fineness, and ability to modify the pore structure of the paste. The incorporation of ceramic waste as a substitute for conventional components can modify the microstructure and thermal behavior of the cementitious material, particularly when it promotes a greater number of air pores. Recent studies on cementitious materials with recycled waste confirmed that the introduction of less dense and/or more porous materials can significantly reduce thermal conductivity [73].

More significant differences were observed when 50% of the natural sand was replaced. The 50NS/50EC mixture, which incorporated expanded clay, had an average thermal conductivity of 1.382 W/(m·K), representing a reduction of 24.70% compared to the control. This result confirms the beneficial effect of lightweight aggregates on thermal insulation.

This reduction is directly related to the highly porous structure of expanded clay. The internal pores contain air, whose thermal conductivity is much lower than that of solid mineral phases, resulting in greater resistance to heat flow. Recent research on mortars with lightweight aggregates has shown that replacing sand with expanded materials increases porosity and significantly reduces thermal conductivity [74,75].

The results obtained for mortars to which BBA-L and BBA-PG were added showed that BBA-L has greater potential as an insulating material. BBA-L had a density of 1.89 g/cm^3^ and a water absorption of 23.8%, while BBA-PG had a higher density of 2.12 g/cm^3^ and a considerably lower water absorption of 11.9%. Therefore, BBA-L has a potentially more porous structure with a greater volume of internal voids, which hinders heat transfer.

This difference was particularly interesting because it demonstrated that it is not enough to consider the percentage of waste substitution to predict thermal behavior; the intrinsic properties of the waste itself are decisive. The lower density and higher water absorption of BBA-L favor a structure with a greater amount of trapped air and, therefore, lower thermal conductivity.

This result is consistent with the work of Cuenca-Moyano et al. (2023) [55], who observed that incorporating BBA from olive-based biomass can reduce the thermal conductivity of concrete by up to 43%, precisely due to the lightweight and porous nature of the waste. More recently, Rosales et al. (2025) [71] confirmed that incorporating BBA as a substitute for aggregates improves thermal insulation properties, resulting in an improvement of approximately 35% in thermal insulation compared to conventional lightweight concrete.

The use of MRA resulted in a smaller reduction than that obtained with EC or BBA, but it remains significant.

Jin et al. (2024)[76] studied the thermal behavior of concrete with recycled aggregates experimentally and numerically and confirmed that the use of recycled aggregates significantly alters the heat transfer pathways within the material. Furthermore, Jin et al. (2025) [76,77] demonstrated through a mesoscale analysis that the presence of pores and old mortar associated with the recycled aggregate increased the material’s thermal resistance.

The results of this study are also consistent with those of Cantero et al. (2020) [72], who found that the simultaneous use of recycled materials as substitutes for cement and aggregates can reduce thermal conductivity, attributing this improvement to reduced density and increased open porosity.

The most pronounced effect on thermal properties was obtained by adding wood waste.

The 50NS/50W mixture had an average thermal conductivity of only 0.751 W/(m·K), representing a 59.07% reduction compared to the control. The mixture containing 10% NaOH-treated wood (90NS/10WT) exhibited an average thermal conductivity of 0.978 W/(m·K), which was 46.72% lower than the control. Therefore, both formulations demonstrated thermal insulation potential far superior to that of conventional mineral mixtures. This performance can be attributed to wood’s low density, cellular structure, and low intrinsic thermal conductivity, as well as the significant amount of air associated with its structure. Wang et al. (2024) [78] observed that the use of wood waste as aggregate significantly improved the thermal insulation properties of recycled concrete, while maintaining adequate mechanical performance through proper optimization of the mix design.

From a thermal standpoint, the results allowed the formulations to be classified into three levels. The mixtures with cement substitution showed relatively small improvements, ranging from approximately 2.4% to 7.3%. The mixtures with sand substitution showed considerably greater improvements, ranging from 10.3% for MRA to 59.1% for wood. Finally, the hybrid formulations showed reductions ranging from approximately 20.5% to 54.5%, depending mainly on the type of waste used.

Consequently, sand substitution had a much more decisive influence on thermal conductivity than cement substitution. Among the wastes studied, wood provided the greatest insulation potential, followed by BBA-L and EC. BBA-PG exhibited intermediate performance, while MRA produced a more moderate reduction in thermal conductivity.

From an application perspective, the BBA-L + EC mixtures proved particularly interesting because they combine a significant reduction in thermal conductivity (38.5%) with a high utilization rate of mineral waste. On the other hand, formulations with wood + EC show the greatest potential for applications where thermal insulation is a priority, achieving reductions of over 47%. Thus, the selection of waste materials must be made by simultaneously considering thermal conductivity, mechanical strength, density, porosity, water absorption, and durability.

For this reason, and given that this study evaluates the use of mortars containing byproducts and analyzes their thermal insulation properties, correlation analyses were conducted between thermal conductivity and UPV, porosity, bulk density, and compressive strength to further investigate the relationship between thermal and mechanical/physical properties. Both Pearson’s and Spearman’s correlation coefficients were calculated. Table 13 below shows the results obtained.

These results indicate that the thermal behavior is closely associated with the physical and mechanical characteristics of the investigated materials. The positive relationship between thermal conductivity and bulk density suggests that denser materials tend to provide more continuous solid paths for heat transfer, whereas the negative correlation with porosity indicates that increasing pore volume reduces heat transfer through the material. The positive correlations with UPV and compressive strength further suggest that microstructural densification, which improves mechanical performance, is also associated with increased thermal conductivity.

**Figure 14:**
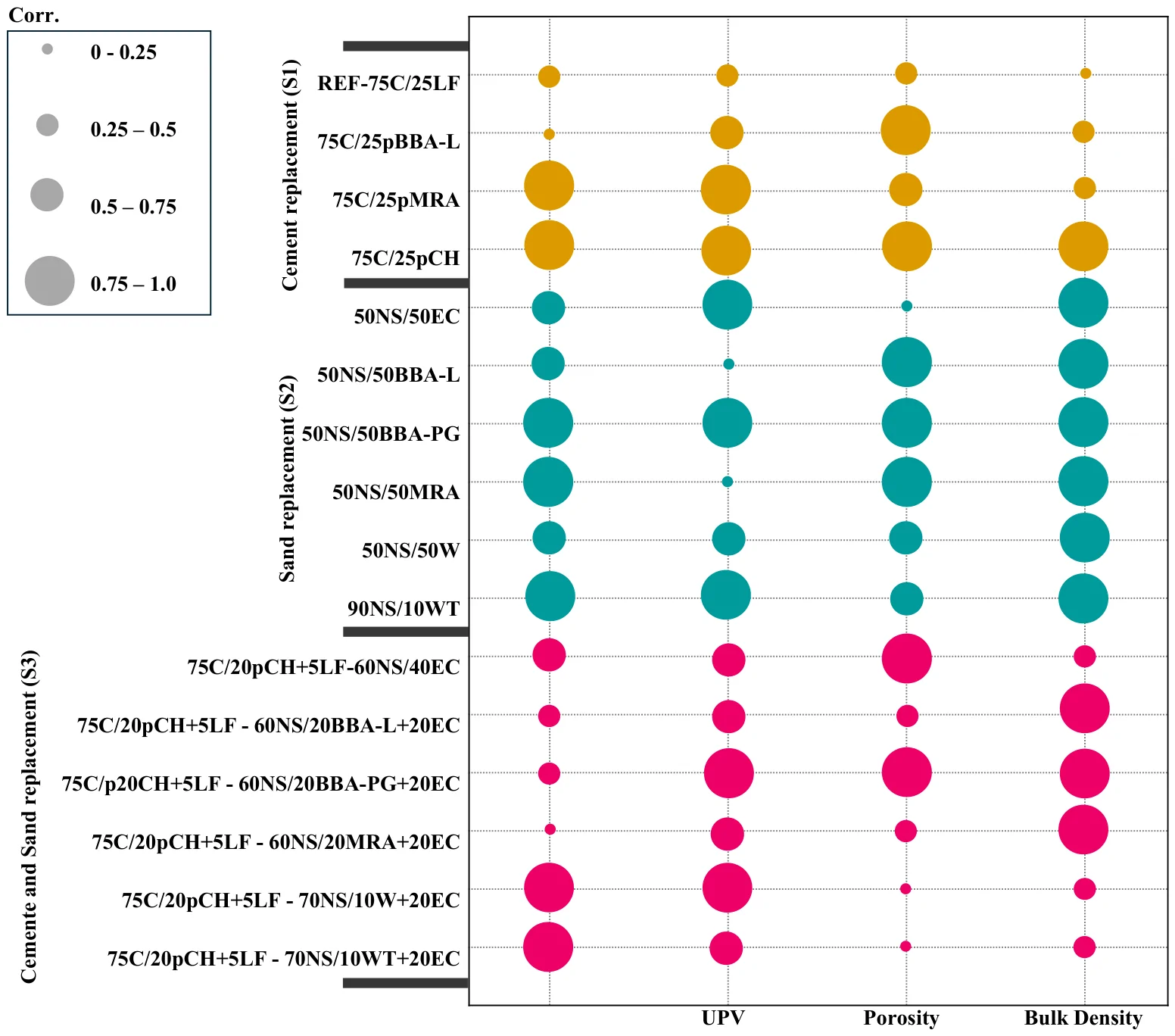
Bubble plot of Pearson correlations between the thermal properties and the physical-mechanical properties of mortars with different substitution strategies.

Figure 14 presents the Pearson correlation analysis between the different variables. The size of the bubbles represents the magnitude of the correlation coefficient, while the colors distinguish between the three waste incorporation strategies: cement substitution (S1), sand substitution (S2), and simultaneous cement and sand substitution (S3). This analysis made it possible to identify which properties had the greatest influence on the thermal behavior of the different families of mortars.

In general terms, the figure showed that the highest correlations were found in porosity, bulk density, and UPV, while the relationship with compressive strength exhibited greater variability depending on the type of substitution. This behavior showed that the thermal response of the mortars was influenced primarily by changes in their internal structure and, in particular, by the number and distribution of pores.

One of the most significant findings in Figure 14 was the close relationship between the thermal properties and the parameters describing the porous structure of the mortar. Density and porosity are, in this regard, two complementary variables.

A decrease in density typically implies an increase in pore volume and, therefore, a greater amount of air within the matrix. As a result, thermal conductivity decreases. This trend was already identified in the independent analysis of density and porosity, where a very high inverse relationship between the two variables was observed (Figure 12). Specifically, the previously obtained experimental correlation was as follows:

Porosity–density for cement replacement: R^2^ = 0.9192.

Porosity–density for sand replacement: R^2^ = 0.8716.

Porosity–density for simultaneous replacement: R^2^ = 0.8530.

Therefore, the Pearson analysis in Figure 14 reinforces the previous interpretation: the reduction in thermal conductivity is fundamentally associated with the creation of a lighter and more porous structure.

The results showed that porosity and density are the parameters most directly related to thermal behavior, while the UPV and compressive strength act as indirect indicators of the degree of compaction of the matrix. It should be noted that the relationship between thermal conductivity and the various properties analyzed was clearly observed in Series 2, in which sand was substituted. However, in Series 1 and Series 3, this relationship was not clearly observed, as the size of the bubbles depicted in the figures is small, indicating a Pearson correlation coefficient of less than 0.25 in many cases.

Among the formulations studied, 75C/20pCH+5LF-60NS/20MRA+20EC exhibited the most balanced mechanical and thermal behavior, as shown in Figure 14, where the ratio between the two properties is less than 0.25. At 90 days, this mixture reached a compressive strength of approximately 39 MPa, a value virtually identical to that of the reference mortar, while its thermal conductivity was reduced to approximately 1.44 W/(m·K), equivalent to a decrease of nearly 22% compared to the control. When compared to mixtures containing materials with lower density, such as wood or BBA-L, it was observed that they provided greater reductions in thermal conductivity, but at the cost of considerable losses in mechanical strength.

### 4.3. Micro-Properties

To evaluate the microstructural properties of the manufactured mortars, an SEM-EDS analysis was performed on four mixtures selected based on their thermal properties: 75C/20pCH+5LF-60NS/20BBA-L+20EC, 75C/20pCH+5LF-60NS/20MRA+20EC, 75C/20pCH+5LF-70NS/10W+20EC and 75C/20pCH+5LF-70NS/10WT+20EC. The samples were fractured by hand to expose a natural fracture surface, oven-dried at 60 °C to constant mass, and stored in a desiccator. They were then mounted on specimen stubs with carbon adhesive tape, coated with carbon, and loaded into a field-emission scanning electron microscope for imaging and EDS analysis.

Points with distinct morphological phases were selected for EDS analysis to identify the phases present and the effect of byproduct addition on matrix formation.

#### SEM Maps

The SEM micrographs of the analyzed mixtures show heterogeneous microstructures in which unhydrated phases, calcium hydration products, and C–S–H- and C–(A)–S–H-type gels coexist. The overall appearance of the matrices reveals significant differences in density, continuity of the hydrated phase, and quality of the interfacial transition zone (ITZ)—aspects that are critical to the material’s mechanical strength and durability [38].

**Figure 15:**
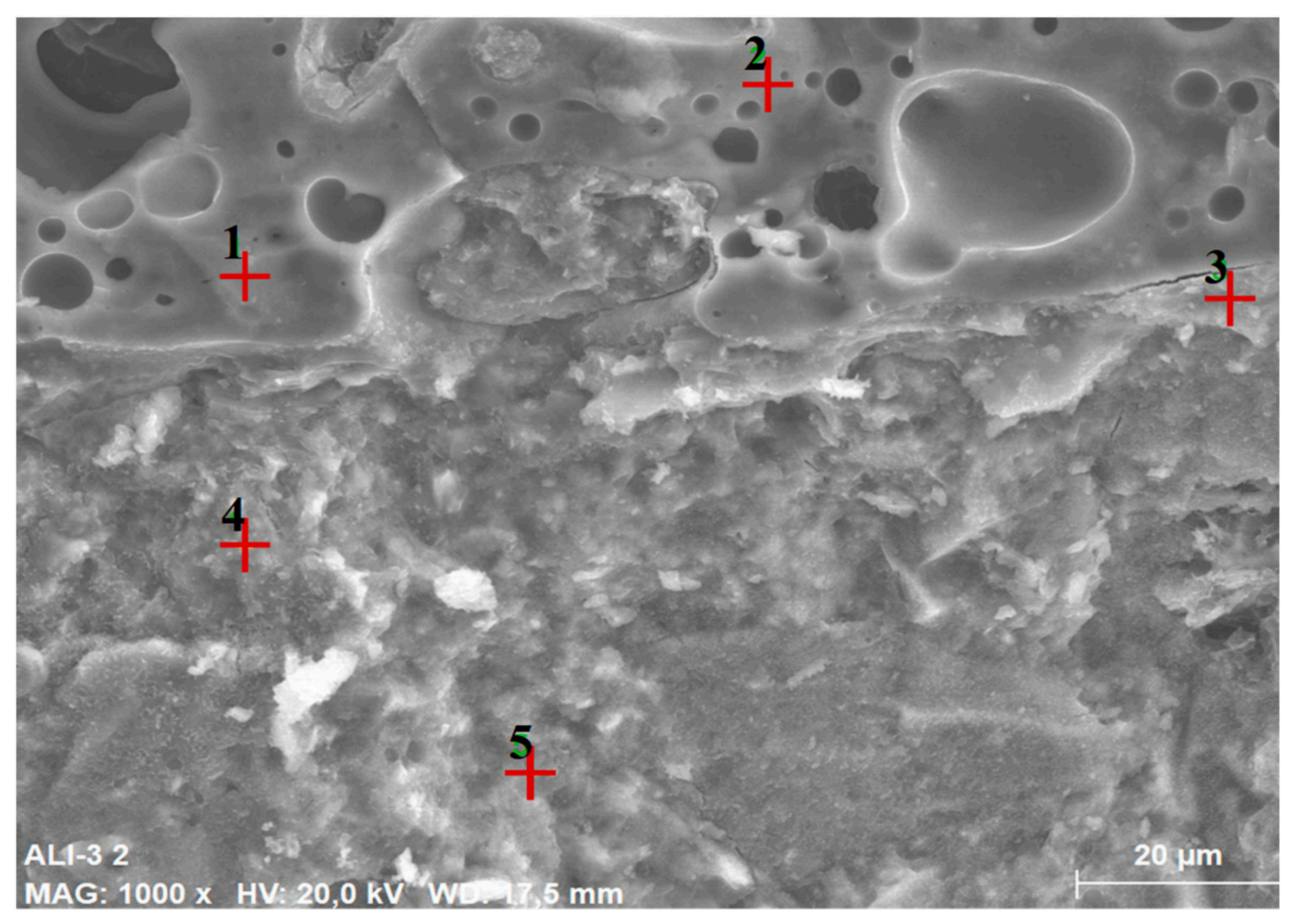
SEM image of 75C/20pCH+5LF-60NS/20MRA+20EC.

Figure 15 shows an image of the 75C/20pCH+5LF-60NS/20MRA+20EC mixture. This mixture exhibited a microstructure in which the unhydrated phases and the advanced hydration products could be distinguished. The matrix appeared relatively dense, with fewer pores and a well-consolidated ITZ, suggesting efficient hydration. This behavior is consistent with that described for cementitious systems incorporating ceramic waste and aluminosilicate materials, in which fine particles can act simultaneously through a filling effect and through pozzolanic reactions, promoting the additional formation of cementitious products and reducing porosity [79,80,81]. In particular, SEM observations have shown that the incorporation of ceramic powder can refine the pore structure and densify the ITZ, reducing the size of the pores and the crystalline hydration products.

At point 1, an unreacted aluminosilicate phase was observed, containing higher amounts of silica (SiO_2_) 45.34%, alumina (Al_2_O_3_) 30.16% and iron oxide (FeO) 10.25%. The high concentration of SiO_2_ and Al_2_O_3_ is characteristic of ceramic materials and other aluminosilicate precursors, whose reactivity depends largely on their degree of amorphization, chemical composition, particle size, and activation or hydration conditions [80,82]. The angular and rough appearance of these particles is also consistent with the characteristic morphology of ceramic waste or chamotte.

In contrast, points 2, 3, 4 and 5 exhibited calcium phases and Ca–Si–Al phases with a more compact morphology, indicating the formation of hydration products associated with portlandite and C–S–H/C–A–S–H-type gels. The simultaneous presence of Ca, Si, and Al is particularly relevant, since the incorporation of aluminosilicate materials into calcium-rich systems can promote the formation of hydrated phases that incorporate aluminum into the C–S–H gel structure, giving rise to C–A–S–H-type products. These processes have been extensively linked to the reaction between sources of silica and alumina and the calcium phases available in the system [38]. Furthermore, in systems containing ceramic waste, it has been observed that reactive silica from this waste can react with Ca(OH)_2_ to generate additional C–S–H, contributing to a more compact microstructure [83]. The observed transition from relatively angular aluminosilicate particles to hydrated products with a more compact morphology is consistent with the general mechanism of dissolution of aluminosilicate precursors and the subsequent formation of cementitious products. The presence of residual particles alongside hydration products is also common in cementitious matrices, since hydration does not necessarily imply the complete reaction of all precursor particles.

The reduced number of pores observed in the matrix may be associated with the progressive development of hydration products, which partially fill the spaces initially occupied by water and reduce the connectivity of the porous network. The formation of C–S–H is one of the main mechanisms responsible for the densification of the cement paste and the development of its mechanical properties. In mixtures containing ceramic waste, this effect may be enhanced by the combined effect of the filling action of fine particles and their pozzolanic activity, which promotes the formation of secondary C–S–H and a denser internal structure [84,85].

In this regard, the microstructure observed in the 75C/20pCH+5LF-60NS/20MRA+20EC mixture appears to indicate a favorable interaction between the aluminosilicate phases and the calcium sources, promoting the formation of hydration products and a progressively more compact matrix.

**Figure 16:**
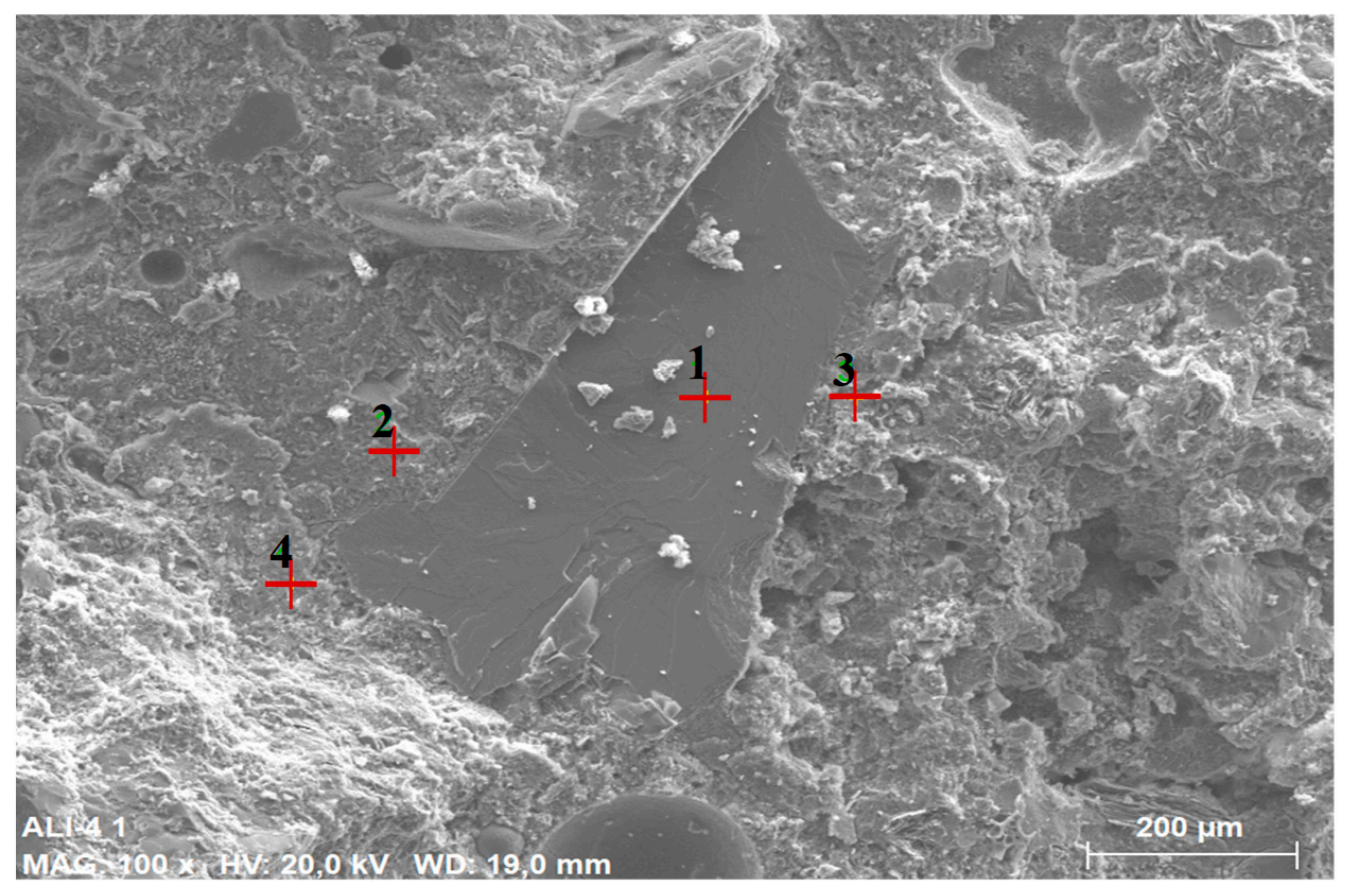
SEM image of 75C/20pCH+5LF-60NS/20BBA-L+20EC.

The SEM image of the 75C20pCH5F20AR20BBAL60NS mixture is shown in Figure 16. A relatively continuous hydrated matrix was observed, with aluminosilicate particles firmly embedded in the paste. At point 1, the high concentration of SiO_2_ (60.7%) and Al_2_O_3_ (21.1%), together with the angular and rough morphology, is consistent with a residual aluminosilicate phase associated with the ceramic materials and BBA. The presence of unreacted or partially reacted particles is common in mortars containing ceramic waste, especially when some of their components exhibit low reactivity. Recent studies have shown that the degree of reaction of these wastes depends on their composition, particle size, and substitution ratio [86,87].

Points 2, 3 and 4 exhibited Ca–Si–Al compositions and a more homogeneous morphology, consistent with C–S–H and C–(A)–S–H type hydration products. The presence of these products may contribute locally to the filling of voids and the consolidation of the matrix. However, this densification observed at the microscopic scale must be interpreted in conjunction with the total porosity of the mortar, which was found to be 33.7%. This value indicates that, although there are areas of good integration between the particles and the matrix, a significant amount of porous space remains in the material as a whole. Studies on mortars containing ceramic waste and biomass bottom ash have shown that the evolution of porosity depends heavily on the proportion and particle size distribution of the waste; a reduction in pores may occur when the filling effect predominates, but also an increase in porosity when there is a high quantity of angular particles or particles with high water absorption [88].

In this regard, the observed microstructure may partially explain the high porosity of 33.7%. The roughness and angularity of the particles promote mechanical anchoring, but they can also hinder efficient packing and create voids around the particles. Furthermore, the incorporation of BBA can increase water absorption and alter the porous structure of the mortar due to its rough surface and high absorption capacity [89].

**Figure 17:**
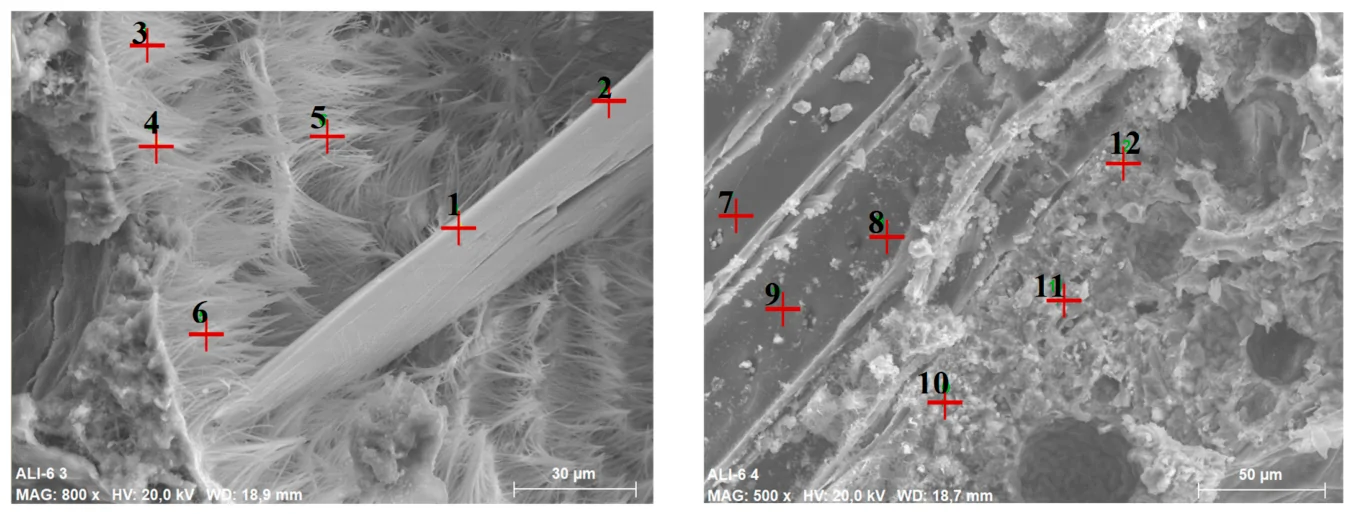
SEM image of 75C/20pCH+5LF-70NS/10W+20EC.

Figure 17 shows the analyzed SEM image corresponding to the 75C/20pCH+5LF-70NS/10W+20EC mixture. The image revealed a heterogeneous microstructure in which hydrated phases, unreacted particles, and wood-derived fibrous elements coexist. This heterogeneity is characteristic of cementitious systems modified with organic and ceramic additives and is consistent with findings reported in previous studies on cement–waste hybrid matrices.

Long wood fibers were observed in the image, accompanied by secondary fibrils. Their elongated morphology, with a rough surface and irregular edges, promotes mechanical adhesion to the matrix, although it also introduces areas of discontinuity and micropores at the interface.

This fiber–matrix interface is critical, as wood can partially inhibit cement hydration due to the presence of extractives, especially when it has not been chemically treated [90]. This is a factor that leads to a reduction in compressive strength.

Image analysis showed that calcium-rich phases, slab-shaped hydration products, and Ca–Si–Al phases coexisted. At points 1, 2 and 12, CaO contents exceeding 89% were obtained, corresponding to regions highly enriched in calcium.

Points 3, 4 and 5 exhibited compositions enriched in Ca, Al, and S, along with a needle-like morphology. This combination is consistent with the formation of sulfoaluminate products, particularly ettringite, which can form during hydration through the reaction between aluminate phases, calcium sources, and available sulfates.

At point 11, the combined presence of Ca, Si, and Al is consistent with the formation of a C–(A)–S–H-type gel. The incorporation of wood waste can modify this process due to the high water absorption capacity of the lignocellulosic particles and the presence of organic compounds that can interact with the cementitious phases. This water absorption is primarily due to the total porosity of the mortar, which reaches 21.86%. This value indicated that the matrix exhibited a noticeable degree of porosity, although considerably lower than that observed in mortars with extremely open structures. Studies on cementitious materials containing lignocellulosic waste have shown that high water absorption and the irregular morphology of the particles can alter the pore distribution and increase the porosity of the hardened material [91].

**Figure 18:**
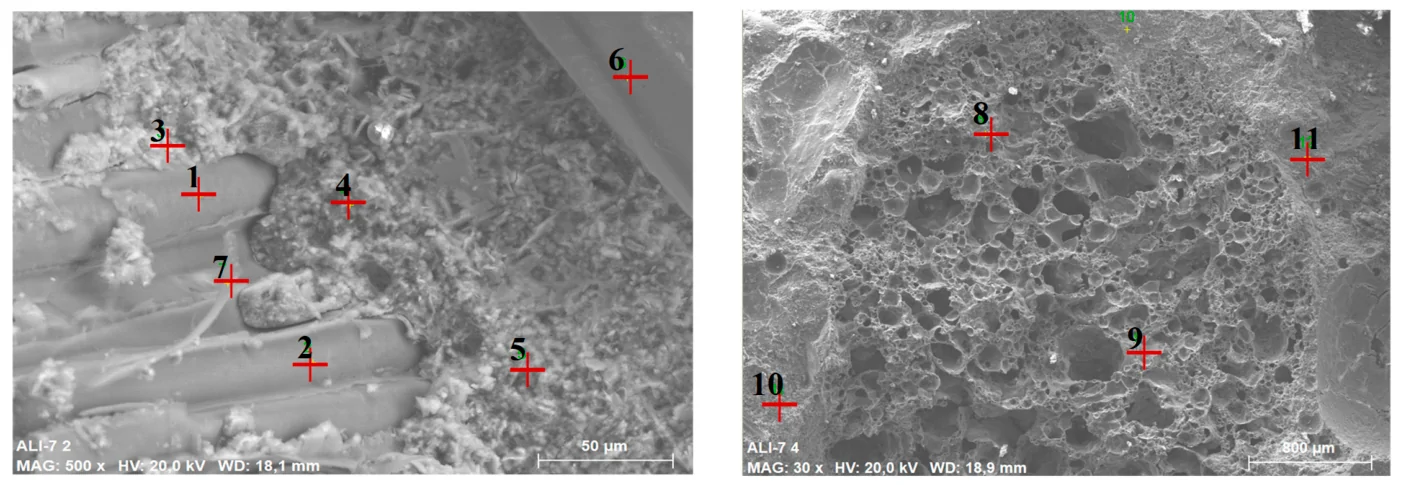
SEM image of 75C/20pCH+5LF-70NS/10WT+20EC.

The SEM image shown in Figure 18 for the 75C/20pCH+5LF-70NS/10WT+20EC sample revealed a heterogeneous microstructure in which calcium phases, hydrated gels, lignocellulosic fibers and partially unreacted aluminosilicate particles coexisted. This combination is characteristic of mortars modified with wood waste and chamotte and is consistent with the EDS results previously described for this mixture made with untreated wood.

The image at the highest magnification, on the left, showed a surface dominated by wood fibers, clearly visible as elongated, flexible structures accompanied by secondary fibrils. The second image, on the right, taken at a lower magnification, showed a region with high porosity, featuring rounded cavities and fracture zones.

Points 2, 6 and 10 have CaO contents greater than 88%, consistent with calcium-rich phases, such as portlandite and/or calcium carbonate. Points 1, 3, 4, 5 and 12 exhibit compositions rich in Ca, Si, and Al, consistent with the formation of C–(A)–S–H gels. These products may be related to both cement hydration and the pozzolanic reaction of the chamotte, whose aluminosilicate fraction can react with the calcium phases and promote the formation of additional hydrated products. Recent studies have shown that ceramic waste can contribute to the formation of C–A–S–H gels and to the densification of the matrix when it contains a sufficiently reactive fraction [53].

The comparison with the 75C/20pCH+5LF-70NS/10W+20EC mixture proved significant. The 75C/20pCH+5LF-70NS/10WT+20EC mixture exhibited a greater number of Ca–Si–Al regions, specifically at points 1, 4, 5 and 12, suggesting greater local development of C–(A)–S–H-type hydration products. This behavior may be related to the pre-treatment of the wood with NaOH, which modifies the surface of the lignocellulosic particles and may partially remove components such as hemicellulose and lignin, thereby increasing the roughness and contact area with the cementitious matrix. Experimental studies have shown that alkaline treatment of wood waste can improve compatibility and interfacial bonding between the wood and the cementitious matrix [92,93]. Therefore, the microstructure of 75C/20pCH+5LF-70NS/10WT+20EC can be interpreted as the result of the combined action of cement hydration, the pozzolanic activity of chamotte, and the improvement of the interface associated with the alkaline treatment of the wood.

## 5. Conclusions

The conclusions drawn from the study are detailed below.

- The incorporation of by-products significantly altered the bulk density and porosity of the mortars, with an inverse relationship observed between these two properties.
- Formulations containing lightweight or highly porous materials exhibited a higher pore ratio, while modifying the cementitious phase with chamotte and limestone filler appeared to improve the packing of the matrix.
- Chamotte performed particularly well as a cement substitute. At 90 days, the 75C/25pCH mixture achieved an approximately 41% increase in compressive strength compared to the reference mortar and a 16.9% improvement in flexural strength at 28 days, attributable to the combination of the filler effect, high fineness, and possible pozzolanic activity of its silico-aluminous phases.
- The evolution of the ultrasonic pulse velocity (UPV) with curing age confirmed the progressive densification and consolidation of the cementitious matrix. A direct relationship was observed between UPV and compressive strength and an inverse relationship with porosity. This relationship was particularly significant in mixtures with sand replacement, where coefficients of determination of R^2^ = 0.991 were obtained for CS–UPV and R^2^ = 0.9719 for UPV–porosity, demonstrating the potential of UPV as a non-destructive technique for indirectly evaluating the quality and evolution of the matrix.
- All formulations containing waste materials showed a reduction in thermal conductivity compared to the reference mortar, with the effect being most pronounced in the sand-substituted mixtures. Cement substitution yielded relatively limited improvements, whereas mixtures containing wood, BBA-L, and expanded clay demonstrated greater insulation potential. These results confirm that the nature, density, and porosity of the incorporated materials are key factors in modifying heat transfer.
- The incorporation of MRA allowed for a favorable compromise between density reduction, mechanical performance, and thermal insulation. In particular, the 75C/20pCH+5LF–60NS/20MRA+20EC formulation achieved approximately 39 MPa at 90 days, practically matching the reference mortar, while reducing thermal conductivity to approximately 1.44 W/(m·K), equivalent to a reduction of about 22%.
- Pearson’s correlation analysis confirmed that the thermal response of mortars is primarily determined by their internal structure. The most significant correlations were observed with porosity and bulk density, while the UPV and compressive strength exhibited a more indirect relationship, as they are linked to the degree of compaction and continuity of the matrix. This trend was particularly clear in the Series 2 mixtures, corresponding to sand substitution, while in Series 1 and 3 the correlations were less pronounced, indicating that the type of substitution modifies the relationship between the mortar’s different properties.

The joint analysis demonstrates that the selection of waste materials for the development of sustainable mortars must take into account their thermal, mechanical, and physical properties simultaneously. Formulations with higher thermal insulation capacity do not necessarily offer the best mechanical performance, since increased porosity and reduced density can improve thermal performance at the cost of decreased strength and altered other material properties. In this regard, thermal properties must be interpreted in conjunction with mechanical strength, density, porosity, and UPV to identify formulations that are truly suitable for construction applications. Among the mixtures studied, the combination of chamotte, MRA, and expanded clay offers a particularly balanced trade-off between thermal and mechanical performance.

## Figures and Tables

**Table 1:** Chemical properties of cement (CEM I 52.5 N).

	SiO_2_	CaO	AL_2_O_3_	Fe_2_O_3_	MgO	SO_3_	TiO_2_	K_2_O	LOI
CEM I 52.5 N	18.12	66.33	4.26	2.89	1.35	5.45	0.18	1.12	0.03

**Table 2:** Properties of the materials.

Properties	LF	NS	EC	BBA-L	BBA-PG	MRA	Wood	
Size (mm)	0–1	0–2	2–10	0–2	0–2	0–2	0–4	UNE-EN 933-1 [24]
Density SSD (Kg/m^3^)	2.49	2.57	0.262	1.897	2.12	2.390	0.96	UNE-EN 1097-6 [25]
Water Absorption (%)	1.12	0.18	34	23.847	11.91	4.760	286.08	
Friability Ratio (%)	-	0.1–2	-	27.5	30.7	24.2	-	UNE-EN 146404 [26]
Soluble Sulphate (%)	0.27	>0.01	>0.4	0.201	0.286	0.06	-	UNE-EN 1744-1 [27]
Chloride Content (%)	0.42	>0.01	>0.1	0.19	2.72	0.09	-	
Organic Matter Content (%)	0.72	-	-	3.4	1.52	0.4	-	
Sand Equivalent (%)	-	-	-	-	-	58	-	UNE-EN 933-8 [28]

**Table 3:** Chemical properties of BBA-L and BBA-PG.

	SiO_2_	CaO	AL_2_O_3_	Fe_2_O_3_	MgO	SO_3_	TiO_2_	K_2_O	LOI
BBA-L	58.20	15.00	10.30	5.46	3.07	0.23	0.74	4.67	2.26
BBA-PG	28.90	38.70	9.05	5.71	4.24	1.15	0.57	8.65	3.24

**Table 4:** Chemical properties of MRA.

	SiO_2_	CaO	AL_2_O_3_	Fe_2_O_3_	MgO	SO_3_	TiO_2_	K_2_O	LOI
MRA	36.57	40.48	9.04	3.73	2.89	3.33	0.48	1.90	0.42

**Table 5:** Properties of alternative by-product materials as partial substitutes for cement.

Properties	pBBA-L	pBBA-PG	pMRA	pCH
Real Density (kg/m^3^)	2438	2312	2520	2374
Blaine fineness (cm^2^/g)	5096	5733	5512	4756
Organic matter content (%)	2.18	1.24	0.8	0.6
Acid-soluble sulphate (% SO_3_)	0.25	0.17	0.95	0.33
Chloride content (%)	0.16	1.12	0.23	0.28
CaO	17.15	29.13	58.54	17.5
SiO_2_	55.39	38.15	15.73	49.9
Al_2_O_3_	10.06	9.12	4.35	17.6
Fe_2_O_3_	5.88	5.73	2.94	6.81
MgO	3.12	3.06	14.56	2.14
K_2_O	4.8	8.91	0.81	3.68

**Table 6:** Tests conducted on mortars. Date of testing, number of test specimens, dimensions, and applicable standards.

Property	Test Method	Curing Time (d)	Types and Number of Specimens	Standard
Mechanical performance	Compressive strength	2, 7, 28, 90	Prismatic 40 × 40 × 80 mm (3 per age)	UNE-EN 196-1
	Flexural strength	2, 7, 28, 90	Prismatic 40 × 40 × 160 mm (3 per age)	UNE-EN 196-1
Physical and Durability properties	Bulk density and porosity	28	Prismatic 40 × 40 × 160 mm (3)	UNE-EN 1015-10 [45]
	Ultrasonic Pulse Velocity (UPV)	28	Prismatic 40 × 40 × 160 mm (3)	UNE-EN 12504-4 [46]
	Thermal properties	28	Prismatic 400 × 400 × 35 mm (3)	ASTM C518 [47]
Micro-properties	SEM maps	28	-	-

**Table 7:** Mortar dosage design. Units in grams.

	Water	W. abs	Cements	SCM				Natural Aggregates		Recycled Aggregates				Wef/C	Slump (cm)
			CEM I	LF	pBBA-L	pMRA	pCH	NS	EC	BBA-L	BBA-PG	MRA	W		
**SERIES 0 Control**															
**CR-100C**	225	-	450	-	-	-	-	1350	-	-	-	--	-	0.5	17
**REF-75C/25LF**	225	-	337.5	112.5	-	-	-	1350	-	-	-	-		0.5	17
**SERIES 1 Cement replacement**															
**75C/25pBBA-L**	225	-	337.5	-	112.5	-	-	1350	-	-	-	-		0.5	16
**75C/25pMRA**	225	-	337.5	-	-	112.5	-	1350	-	-	-	-		0.5	17
**75C/25pCH**	225	-	337.5	-	-	-	112.5	1350	-	-	-	-			
**SERIES 2 Sand replacement**															
**50NS/50EC**	225	-	337.5	112.5	-	-	-	675	69.3	-	-	-	-	0.5	16
**50NS/50BBA-L**	225	139	337.5	112.5	-	-	-	675	-	500.2	-	-	-	0.55	17
**50NS/50BBA-PG**	225	122	337.5	112.5	-	-	-	675	-	-	611.7	-	-	0.6	16
**50NS/50MRA**	225	69	337.5	112.5	-	-	-	675	-	-	-	631.1	-	0.58	17
**50NS/50W**	225	714	337.5	112.5	-	-	-	675	-	-	-	-	248.8	0.6	16
**90NS/10WT**	225	103.5	337.5	112.5	-	-	-	1215	-	-	-	-	59 (WT)	0.5	16
**SERIES 3 Combination Cement and Sand replacement**															
**75C/20 p CH+5LF-60NS/40EC**	225	-	337.5	22.5	-	-	90	810	55.5	-	-	-	-	0.5	16
**75C/20 p CH+5LF- 60NS/20BBA-L+20EC**	225	63	337.5	22.5	-	-	90	810	27.7	200.8	-	-	-	0.54	17
**75C/20 p CH+5LF-60NS/20BBA-PG+20EC**	225	45	337.5	22.5	-	-	90	810	27.7	-	244.6	-	-	0.54	16
**75C/20 p CH+5LF-60NS/20MRA+20EC**	225	14	337.5	22.5	-	-	90	810	27.7	-	-	252.4	-	0.51	16
**75C/20 p CH+5LF-70NS/10W+20EC**	225	25	337.5	22.5	-	-	90	945	27.7	-	-	-	49.8	0.50	16
**75C/20 p CH+5LF-70NS/10WT+20EC**	225	18	337.5	22.5	-	-	90	945	27.7	-	-	-	59 (WT)	0.50	16

**Table 8:** Variation in long-term compressive strength (90 days) compared to the reference.

**SERIES 0 Control**	
REF-75C/25LF	-
**SERIES 1 Cement replacement**	
75C/25pBBA-L	+7.3%
75C/25pMRA	+8.7%
75C/25pCH	+40.96%
**SERIES 2 Sand replacement**	
50NS/50EC	−5.9%
50NS/50BBA-L	−53.7%
50NS/50BBA-PG	−34.2%
50NS/50MRA	−8.7%
50NS/50W	−92.4%
90NS/10WT	−58.2%
**SERIES 3 Combination Cement and Sand replacement**	
75C/20pCH+5LF-60NS/40EC	+26.4%
75C/20pCH+5LF-60NS/20BBA-L+20EC	−41.7%
75C/20pCH+5LF-60NS/20BBA-PG+20EC	−23.2%
75C/20pCH+5LF-60NS/20MRA+20EC	≈0%
75C/20pCH+5LF-70NS/10W+20EC	−74.7%
75C/20pCH+5LF-70NS/10WT+20EC	−51.3%

**Table 9:** 2-, 7-, 28-, and 90-day flexural strength and strength variation at 28 days compared to reference.

Flexural Strength (MPa)					
	2D	7D	28D	90D	Δ 28D (%)
**SERIES 0 Control**					
**CR-100C**	7.12 ± 0.11	7.93 ± 0.21	8.11 ± 0.17	8.93 ± 0.08	
**REF-75C/25LF**	6.67 ± 0.12	6.69 ± 0.09	6.79 ± 0.08	8.20 ± 0.11	-
**SERIES 1 Cement replacement**					
**75C/25pBBA-L**	5.73 ± 0.12	6.53 ± 0.09	6.81 ± 0.14	7.03 ± 0.16	+0.3%
**75C/25pMRA**	4.93 ± 0.13	6.32 ± 0.08	6.97 ± 0.12	7.15 ± 0.18	+2.7%
**75C/25pCH**	6.4 ± 0.11	6.8 ± 0.16	7.94 ± 0.11	8.98 ± 0.08	+16.9%
**SERIES 2 Sand replacement**					
**50NS/50EC**	6.18 ± 0.06	6.63 ± 0.08	6.73 ± 0.05	6.94 ± 0.09	−0.9%
**50NS/50BBA-L**	3.27 ± 0.09	3.54 ± 0.12	4.66 ± 0.06	4.81 ± 0.05	−31.4%
**50NS/50BBA-PG**	3.77 ± 0.11	5.89 ± 0.06	6.34 ± 0.08	6.53 ± 0.06	−6.6%
**50NS/50MRA**	5.31 ± 0.04	5.85 ± 0.04	7.59 ± 0.10	7.81 ± 0.08	+11.8%
**50NS/50W**	0.57 ± 0.12	0.91 ± 0.12	1.82 ± 0.11	1.84 ± 0.10	−73.2%
**90NS/10WT**	4.06 ± 0.11	4.84 ± 0.13	5.01 ± 0.09	5.87 ± 0.13	−26.2%
**SERIES 3 Combination Cement and Sand replacement**					
**75C/20pCH+5LF-60NS/40EC**	6.11 ± 0.15	6.46 ± 0.12	7.57 ± 0.08	7.92 ± 0.08	+11.5%
**75C/20pCH+5LF-60NS/20BBA-L+20EC**	4.26 ± 0.05	4.83 ± 0.08	5.96 ± 0.12	6.34 ± 0.06	−12.2%
**75C/20pCH+5LF-60NS/20BBA-PG+20EC**	5.64 ± 0.03	6.09 ± 0.07	7.12 ± 0.09	7.3 ± 0.18	+4.9%
**75C/20pCH+5LF-60NS/20MRA+20EC**	6.28 ± 0.08	6.63 ± 0.10	7.45 ± 0.16	8.25 ± 0.12	+9.7%
**75C/20pCH+5LF-70NS/10W+20EC**	2.31 ± 0.09	2.34 ± 0.12	2.79 ± 0.11	3.63 ± 0.07	−58.9%
**75C/20pCH+5LF-70NS/10WT+20EC**	1.98 ± 0.12	4.11 ± 0.17	5.62 ± 0.14	5.79 ± 0.13	−17.2%

**Table 10:** Bulk density and porosity of mortars.

	Bulk Density (g/cm^3^)	Porosity %
**SERIES 0 Control**		
**REF-75C/25LF**	2.06 ± 0.02	14.86 ± 0.05
**SERIES 1 Cement replacement**		
**75C/25pBBA-L**	2.01 ± 0.03	17.09 ± 0.04
**75C/25pMRA**	2.03 ± 0.03	16.98 ± 0.08
**75C/25pCH**	2.01 ± 0.02	17.07 ± 0.01
**SERIES 2 Sand replacement**		
**50NS/50EC**	1.76 ± 0.03	20.10 ± 0.05
**50NS/50BBA-L**	1.48 ± 0.04	33.72 ± 0.63
**50NS/50BBA-PG**	1.77 ± 0.06	24.70 ± 0.31
**50NS/50MRA**	1.94 ± 0.04	21.53 ± 0.43
**50NS/50W**	1.16 ± 0.04	64.12 ± 1.01
**90NS/10WT**	1.69 ± 0.03	26.10 ± 0.55
**SERIES 3 Combination Cement and Sand replacement**		
**75C/20pCH+5LF-60NS/40EC**	1.97 ± 0.03	15.23 ± 0.44
**75C/20pCH+5LF-60NS/20BBA-L+20EC**	1.74 ± 0.03	21.48 ± 0.64
**75C/20pCH+5LF-60NS/20BBA-PG+20EC**	1.86 ± 0.04	16.60 ± 0.42
**75C/20pCH+5LF-60NS/20MRA+20EC**	1.96 ± 0.02	15.73 ± 0.13
**75C/20pCH+5LF-70NS/10W+20EC**	1.34 ± 0.04	21.86 ± 0.85
**75C/20pCH+5LF-70NS/10WT+20EC**	1.56 ± 0.03	25.20 ± 1.47

**Table 11:** 7-, 28- and 90-day UPV values.

UPV (m/s)								
Mix	2D	Δ 2D (%)	7D	Δ 7D (%)	28D	Δ 28D (%)	90D	Δ 90D (%)
**SERIES 0 Control**								
**REF-75C/25LF**	3802 ± 13.12	-	3947 ± 14.01	-	4264 ± 21.12	-	4378 ± 23.54	-
**SERIES 1 Cement replacement**								
**75C/25pBBA-L**	3872 ± 19.21	1.8	3943 ± 23.15	−0.1	4225 ± 12.18	−0.9	4347 ± 26.87	−0.7
**75C/25pMRA**	3887 ± 10.12	−2.2	4206 ± 19.87	2.0	4419 ± 21.54	3.6	4513 ± 15.98	3.1
**75C/25pCH**	3725 ± 9.84	−2.0	3978 ± 16.12	0.8	4487 ± 19.13	5.2	4536 ± 18.96	3.6
**SERIES 2 Sand replacement**								
**50NS/50EC**	3721 ± 23.15	−2.1	3843 ± 19.54	2.6	3952 ± 17.12	7.3	4021 ± 22.19	−8.2
**50NS/50BBA-L**	2641 ± 19.12	−30.5	2826 ± 23.15	−28.4	3054 ± 18.11	28.4	3098 ± 28.16	−29.2
**50NS/50BBA-PG**	3415 ± 24.56	−10.2	3502 ± 18.15	−11.3	3619 ± 21.56	−15.1	3687 ± 23.12	−15.8
**50NS/50MRA**	2987 ± 23.56	−21.4	3381 ± 17.15	−14.3	3536 ± 16.23	−17.1	3669 ± 21.24	−16.2
**50NS/50W**	1259 ± 18.12	−66.9	1521 ± 21.01	−61.5	1638 ± 16.57	−61.	2102 ± 19.85	−52.0
**90NS/10WT**	3051 ± 13.10	−19.8	3178 ± 24.11	−19.5	3312 ± 17.64	−22.3	3375 ± 27.15	−22.9
**SERIES 3 Combination Cement and Sand replacement**								
**75C/20pCH+5LF-60NS/40EC**	3798 ± 21.12	−0.1	3915 ± 24.58	−0.8	4064 ± 23.15	−4.7	4112 ± 19.56	−6.1
**75C/20pCH+5LF-60NS/20BBA-L+20EC**	3235 ± 19.54	−14.9	3367 ± 17.12	−14.7	3416 ± 26.69	−19.9	3472 ± 27.15	−20.7
**75C/20pCH+5LF-60NS/20BBA-PG+20EC**	3441 ± 21.15	−9.5	3506 ± 24.74	−11.2	3657 ± 21.06	−14.2	3944 ± 19.54	−9.9%
**75C/20pCH+5LF-60NS/20MRA+20EC**	3534 ± 19.84	−7.0	3771 ± 26.12	−4.5	3820 ± 25.14	−10.4	3936 ± 21.08	−10.1
**75C/20pCH+5LF-70NS/10W+20EC**	2387 ± 26.12	−37.2	2482 ± 21.45	−37.1	2738 ± 27.13	−35.8	2839 ± 22.36	−35.2
**75C/20pCH+5LF-70NS/10WT+20EC**	3045 ± 19.84	−19.9	3145 ± 23.01	−20.3	3298 ± 26.12	−22.7	3396 ± 21.57	−22.4

**Table 12:** Thermal properties and variation compared to the control mortar.

Mix	Thermal Properties [14–16 °C] (W/m·k)	Thermal Properties [29–31 °C] (W/m·k)	Thermal Properties [39–41 °C] (W/m·k)	Thermal Conductivity [Average] (W/m·K)	Δ Respect CR (%)
**SERIES 0 Control**					
**REF-75C/25LF**	1.821 ± 0.01	1.841 ± 0.02	1.845 ± 0.03	1.836 ± 0.04	-
**SERIES 1 Cement replacement**					
**75C/25pBBA-L**	1.784 ± 0.04	1.791 ± 0.02	1.802 ± 0.01	1.792 ± 0.03	−2.36
**75C/25pMRA**	1.735 ± 0.02	1.746 ± 0.03	1.751 ± 0.03	1.744 ± 0.02	−4.99
**75C/25pCH**	1.692 ± 0.03	1.703 ± 0.01	1.709 ± 0.04	1.701 ± 0.03	−7.32
**SERIES 2 Sand replacement**					
**50NS/50EC**	1.362 ± 0.02	1.387 ± 0.06	1.398 ± 0.05	1.382 ± 0.06	−24.70
**50NS/50BBA-L**	1.415 ± 0.01	1.421 ± 0.04	1.433 ± 0.02	1.423 ± 0.03	−22.48
**50NS/50BBA-PG**	1.532 ± 0.05	1.546 ± 0.02	1.561 ± 0.06	1.546 ± 0.06	−15.76
**50NS/50MRA**	1.624 ± 0.02	1.657 ± 0.05	1.661 ± 0.04	1.647 ± 0.05	−10.26
**50NS/50W**	0.742 ± 0.04	0.753 ± 0.02	0.759 ± 0.05	0.751 ± 0.04	−59.07
**90NS/10WT**	0.971 ± 0.04	0.979 ± 0.01	0.984 ± 0.02	0.978 ± 0.05	−46.72
**SERIES 3 Combination Cement and Sand replacement**					
**75C/20pCH+5LF-60NS/40EC**	1.453 ± 0.06	1.459 ± 0.04	1.464 ± 0.06	1.459 ± 0.06	−20.54
**75C/20pCH+5LF-60NS/20BBA-L+20EC**	1.069 ± 0.06	1.151 ± 0.02	1.168 ± 0.08	1.129 ± 0.05	−38.48
**75C/20pCH+5LF-60NS/20BBA-PG+20EC**	1.273 ± 0.04	1.309 ± 0.01	1.313 ± 0.07	1.298 ± 0.07	−29.27
**75C/20pCH+5LF-60NS/20MRA+20EC**	1.426 ± 0.02	1.432 ± 0.02	1.455 ± 0.06	1.438 ± 0.08	−21.68
**75C/20pCH+5LF-70NS/10W+20EC**	0.829 ± 0.01	0.834 ± 0.04	0.843 ± 0.05	0.835 ± 0.04	−54.49
**75C/20pCH+5LF-70NS/10WT+20EC**	0.923 ± 0.03	0.977 ± 0.05	1.014 ± 0.07	0.971 ± 0.02	−47.09

**Table 13:** Pearson and Spearman correlation coefficients between thermal conductivity and the physical and mechanical properties of the mixtures.

Thermal Conductivity Correlation	Pearson r	*p*	Spearman ρ	*p*
**UPV**	0.834	<0.001	0.828	<0.001
**Porosity**	−0.581	<0.001	−0.550	<0.001
**Bulk density**	0.864	<0.001	0.881	<0.001
**Compressive Strength**	0.804	<0.001	0.724	<0.001

Thermal conductivity showed a strong positive correlation with UPV (Pearson r = 0.834, *p* < 0.001; Spearman ρ = 0.828, *p* < 0.001) and bulk density (Pearson r = 0.864, *p* < 0.001; Spearman ρ = 0.881, *p* < 0.001). A strong positive association was also observed between thermal conductivity and compressive strength (Pearson r = 0.804, *p* < 0.001; Spearman ρ = 0.724, *p* < 0.001). In contrast, thermal conductivity was negatively correlated with porosity (Pearson r = −0.581, *p* < 0.001; Spearman ρ = −0.550, *p* < 0.001).

## Data Availability

The original contributions presented in this study are included in the article. Further inquiries can be directed to the corresponding author.

## Abbreviations

The following abbreviations are used in this manuscript:

MRA	Mixed Recycled Aggregates
BBA-PG	Biomass Bottom Ash—Puente Genil
BBA-L	Biomass Bottom Ash—Linares
W	Wood
WT	Wood Treated
CH	Chamotte
XRF	X-ray fluorescence
LF	Limestone filler
NS	Natural Sand
EC	Expanded Clay
pMRA	Powder Mix Recycled Aggregates
pBBA-PG	Powder Biomass Bottom Ash—Puente Genil
pBBA-L	Powder Biomass Bottom Ash—Linares
pCH	Powder Chamotte
SCM	Supplementary Cementitious Materials
UPV	Ultrasonic Pulse Velocity
CS	Compressive Strength
FS	Flexural Strength
P	Porosity
